# Supplementary material for: A new phylogenetic analysis of Phytosauria (Archosauria: Pseudosuchia) with the application of continuous and geometric morphometric character coding
Source: PeerJ. 2018 Dec 10;6:e5901. doi: 10.7717/peerj.5901 (PMC6292387; doi:10.7717/peerj.5901)
Supplement: Supplemental Information 1 [file peerj-06-5901-s001.docx]

**Appendix 1: Taxonomic list**

**Institutional abbreviations**

**AMNH**: American Museum of Natural History, New York, USA; **BSPG**: Bayerische Staatssammlung für Paläontologie und Geologie, Munich, Germany; **CMNH**: Carnegie Museum of Natural History, Pittsburgh, USA; **FMNH**: Field Museum of Natural History, Chicago, USA; **GPIT**: Institut für Geologie und Paläontologie Tübingen, Tübingen, Germany; **GR**: Ruth Hall Museum of Paleontology, Ghost Ranch, Abiquiu, USA; **ISI**: Indian Statistical Institute, Kolkata, India; **MB**: Museum für Naturkunde, Berlin, Germany; **MBSN**: Museo Civico di Scienze naturali ‘E. Caffi’, Bergamo, Italy; **MCZ**: Harvard University Museum of Comparative Zoology, Cambridge, USA; **MNA**: Museum of Northern Arizona, Flagstaff, USA; **MNHN**: Muséum National d’Histoire Naturelle, Paris, France; **MSM**: Arizona Museum of Natural History (previously Mesa Southwest Museum), Mesa, USA; **MU**: University of Missouri, Columbia, Missouri, USA; **NHMUK**: Natural History Museum, London, UK; **NHMW**: Naturhistorisches Museum Wien, Vienna, Austria; **NMC**: Naturkundemuseum Coburg, Coburg, Germany; **NMMNHS**: New Mexico Museum of Natural History and Science, Albuquerque, USA; **OMNH**: Oklahoma Museum of Natural History, Norman, USA; **PEFO**: Petrified Forest National Park, Arizona, USA; **PPHM**: Panhandle Plains Historical Museum West Texas A&M University, Canyon, USA; **SMF**: Forschungsinstitut und Natur-Museum Senckenberg, Frankfurt/Main, Germany; **SMNS**: Staatliches Museum für Naturkunde Stuttgart, Stuttgart, Germany; **TMM**: Texas Memorial Museum, Austin, USA; **TTU-P**: Museum of the University of Texas Tech, Lubbock, USA; **UCM**: University of Colorado Museum, Boulder, USA; **UCMP**: University of California Museum of Paleontology, Berkeley, USA; **UMMP**: University of Michigan Museum of Paleontology, Ann Arbor, USA; **UMNH**: Natural History Museum of Utah, Salt Lake City, USA; **USNM**: National Museum of Natural History, Washington D.C., USA; **UW**: University of Wisconsin Geological Museum, Madison, USA; **YPM**: Yale Peabody Museum, New Haven, USA; **ZMNH**: Zhejiang Museum of Natural History, Hangzhou, China; **ZPAL**: Instytut Paleobiologii PAN, Warsaw, Poland.

***Diandongosuchus fuyuanensis*** Li *et al.*, 2012

**Age:** Ladinian (*c.* 242–237 Mya)

**Occurrences:** Zhuganpo Member, Falang Formation, southeast Fuyuan County, Yunnan Province, China

**Holotype:** ZMNH M8770, largely complete skeleton missing many caudal vertebrae

**Previously Referred Specimens:** Additional specimen under preparation (personal communication with X.-C. Wu cited by Griffin *et al.* [2017])

**Specimen(s) Used for Scoring:** ZMNH M8770

**Key References:** Li *et al.* (2012); Stocker *et al.* (2017)

**Most Recent Diagnosis:**

Stocker *et al.* (2017) distinguished *D. fuyuanensis* from all other phytosaurs using the following characters: 1) Anterodorsal (nasal) process of premaxilla extending well posterior of external naris; 2) Presence of a fossa expanded in anteroventral corner of external naris; 3) Jugal with pronounced longitudinal ridge on lateral surface; 4) Anterior process of jugal much broader than the posterior process underlying anterior process of quadratojugal; 5) Premaxilla with nine teeth; 6) More than one set of paramedian osteoderms dorsal to the cervical series.

**Comments:** *D. fuyuanensis* was originally described as a rauisuchian archosaur (Li *et al*., 2012), and was subsequently reassessed as the most basal phytosaur (Stocker *et al*., 2017), using the following characters: 1) Posterodorsal process of premaxilla strongly sutured to maxilla; 2) More than six premaxillary teeth; 3) Facial portion of maxilla anterior to anterior edge of antorbital fenestra equal in length or longer than portion posterior to anterior edge of fenestra; 4) Entire anterior margin of scapula straight/convex or partially concave; 5) Anterior portion of coracoid distinctly hooked; 6) Ectepicondylar flange of humerus present; 7) Obturator foramen of the pubis modified into a notch that opens medioventrally; 8) medial side of distal tarsal 4 with foramen/foramina; 9) Articular surface for the fibula on the calcaneum convex and hemicylindrical shaped; 10) Osteoderms covering the appendages; 11) Retroarticular process of the articular and surangular well ventral to the articulation with the quadrate; 12) Lateral margin of the humerus straight from midshaft to proximal portion. The hypothesis that *D. fuyuanensis* is the most basal known phytosaur was not explicitly tested by Stocker *et al*. (2017); its position within Phytosauria is assessed for the first time in this study.

***Wannia scurriensis*** (Langston, 1949)

**Age:** late Carnian–early Norian (*c.* 232–225 Mya)

**Occurrences:** Camp Springs Formation, Dockum Group, Scurry County, Texas, USA

**Holotype:** TTU-P 00539, partial skull

**Previously Referred Specimens:** TTU-P 11422 (Stocker 2010)

**Specimen(s) Used for Scoring:** TTU-P 00539

**Key References:** Langston (1949); Stocker (2013)

**Most Recent Diagnosis:** Stocker (2013) diagnosed *Wannia scurriensis* on the basis of the following proposed autapomorphies: 1) Basitubera that are widely separated mediolaterally; 2) Presence of a ridge on the lateral surface of the jugal; 3) Presence of a thickened shelf along the posteroventral edge of an expanded pterygoid-quadrate wing; 4) ‘Septo-maxillae’ that do not contact one another and do not form part of the internarial septum; 5) Presence of a nasal swelling posterior to the posterior border of the nares. In addition to these autapomorphies, Stocker (2013) also provided a differential diagnosis outlining non-autapomorphic characters that distinguish *W. scurriensis* from other phytosaurs.

**Modified Diagnosis:**  Here, we diagnose *Wannia scurriensis* using the following unique combination of characters: 1) Presence of an anteroposteriorly oriented ridge on the lateral surface of the jugal, positioned toward the middle or ventral edge of the jugal posterior process; 2) Presence of a nasal swelling posterior to the posterior border of the nares; 3) Posterior rim of nares smooth (not rugose); 4) Absence of paired depressions posterior to the posterior narial rim.

**Comments:** Some of the characters (1, 2, 3, 5) used by Stocker (2013) to diagnose *Wannia scurriensis* also occur in species of *Parasuchus* and cannot, therefore, be considered autapomorphic. As noted by Stocker (2013), the separation of the basitubera (character 1) is the same as that in *Parasuchus angustifrons*; however, Stocker suggested that the basitubera in *Parasuchus angustifrons* are not as rounded as those of *W. scurriensis*. Based on our examination of specimens of both taxa, this distinction seems subjective and questionable, particularly given that the holotype of *Parasuchus angustifrons* (BSPG 1931 X 502) has been subjected to dorsoventral compression. Stocker (2013) also noted that the narial swelling (charater 5) is also present in *Parasuchus angustifrons* (and is, therefore, also not autapomorphic), although the latter species can be differentiated from *W. scurriensis* as it possesses paired depressions just posterior to the external nares and a rugose posterior narial rim (BSPG 1931 X 502; Butler *et al*. 2014).

The thickened pterygoid-quadrate shelf (character 3) appears to also be present in TMM 31100-101, a specimen referable to *Parasuchus bransoni* on the basis of its narial morphology (see below). Moreover, the majority of the quadrate is missing in *W. scurriensis*, making character 3 difficult to assess. There are difficulties in scoring character 4 accurately for *W. scurriensis* because interpretative lines have been incorrectly drawn onto the specimen to supposedly mark the positions of the ‘septo-maxillae’, and the dorsal surface of the septum is damaged. On close side-by-side inspection of the holotype and a cast (TTU-P 14911, which lacks the interpretative lines), the septomaxillae do seem to form a midline contact, and do not therefore differ in any significant regard from those of *Parasuchus hislopi*, *Parasuchus bransoni* and *Parasuchus angustifrons*.

An additional partial skull (TTU-P 11422) was considered referable to *W. scurriensis* by Stocker (2010); however, as noted by Stocker (2013), no characters are preserved that would allow this specimen to be diagnosed, and as such this specimen should be classified as an indeterminate parasuchid excluded from Mystriosuchinae.

***Parasuchus bransoni*** (Williston, 1904)

**Age:** late Carnian–early Norian (*c.*  232–225 Mya)

**Occurrences:** Popo Agie Formation, Chugwater Group, Wyoming, USA; Colorado City Formation, Dockum Group, Texas, USA

**Holotype:** FMNH UC 632, skull

**Previously Referred Specimens:** TTU-P 00539 (holotype of *Wannia scurriensis*); TMM 31025-172; TMM 31100-8; TMM 31100-101; TMM 31100-175; TMM 31100-418; TMM 31100-419; TMM 31100-453; TMM 31185-11; TMM 31185-38 (Long & Murry, 1995)

**Specimen(s) Used for Scoring:** FMNH UC 632; TMM 31100-101; TMM 31100-418 and 419 (these two specimens likely represent an associated skull and mandible).

**Key References:** Williston (1904); Lees (1906); Long & Murry (1995); Butler *et al.* (2014); Kammerer *et al.* (2015)

**Most Recent Diagnosis:** Long & Murry (1995) diagnosed *Parasuchus bransoni* with the following combination of characters: 1) Skull low; 2) Orbit directed dorsolaterally; 3) Rostrum moderately long, prenarial length equals postnarial length; 4) Maxilla with straight ventral margin; 5) Posterior portion of maxilla not flared laterally; 6) Interpterygoid vacuity small or absent; 7) Homodont dentition; 8) Alveoli circular throughout; 9) Posterior premaxillary alveolae normal; 10) Upper dentition with 36–45 teeth.

**Modified Diagnosis:** A combination of the three *Parasuchus* characters identified by Butler *et al.* (2014) and Kammerer *et al.* (2015) and one character of Kammerer *et al*. (2015) to distinguish *Parasuchus bransoni* from *Parasuchus hislopi* and *Parasuchus angustifrons*: 1) Presence of an anterior frontal depression; 2) Bifurcated lateral ridge on the squamosal; 3) Row of nodes on the lateral surface of the jugal; 4) Possession of high ‘triangular’ nares.

**Comments:** Long & Murry (1995) used a combination of nine characters to diagnose *Parasuchus bransoni*. Characters such as their 1, 2, 5, 7 and 9 have been criticized for being subjective, vague and prone to taphonomic distortion (Hungerbühler, 1998; Stocker, 2010). Character 6 appears to have been generated based on the distorted morphology of the holotype. All non-Mystriosuchinae members of Parasuchidae have an interpterygoid vacuity which extends anteroposteriorly along at least 50% of the palatal vault, and which only becomes restricted in more derived taxa. This proposed diagnostic character is actually therefore plesiomorphic for Mystriosuchinae. Character 7 is problematic as the assessment of dentition used by Long & Murry (1995) was based on empty alveolae and, therefore, may be unreliable. In any case, some degree of heterodonty has now been recognized in all phytosaurs. Character 9 (here inferred to relate to the increase in size of the posterior two or three premaxillary alveolae in many taxa) is also problematic; although the increase in posterior premaxillary alveolar size is subtler in *Parasuchus bransoni* than in many taxa, it does still occur. Several of the characters) are probably plesiomorphic for Phytosauridae (3, 4, 5, 7, 8, and probably 9) and/or are widespread (1, 2, 8, 10) in basal members of the clade*.*

***Parasuchus hislopi*** Lydekker, 1885

**Age:** late Carnian–early Norian (*c.* 232–225 Mya)

**Occurrences:** Lower Maleri Formation, Pranhita Godavari Valley, Telangana, India; Tiki Formation, Madhya Pradesh, India

**Holotype:** ISI R 42 (neotype), almost complete skeleton including cranium and mandible.

**Previously Referred Specimens:** ISI R 43; ISI R 44; ISI R 45; ISI R 46; ISI R 47; ISI R 160; ISI R 161 (Chatterjee, 1978)

**Specimen(s) Used for Scoring:** ISI R 42; ISI R 43

**Key References:** Lydekker (1885); Chatterjee (1974, 1978); Kammerer *et al.* (2015)

**Most Recent Diagnosis:** Kammerer *et al*. (2015) diagnosed *Parasuchus hislopi* as follows: a species of *Parasuchus* that is 1) distinguished from *Parasuchus bransoni* by a relatively low narial eminence with a raised, rugose posterior margin of the naris (a ‘narial rim’); 2) Distinguished from *Parasuchus angustifrons* by the absence of paired depressions on the anterior portions of the nasals; 3) Tentatively distinguished from ‘*Paleorhinus magnoculus’* by the posterior confluence of the raised margins of the nares.

***Parasuchus angustifrons*** (Kuhn, 1936)

**Age:** late Carnian (*c.* 232–228 Mya)

**Occurrences:** Blasensandstein (lateral equivalent of Hassberge Formation), Middle Keuper Subgroup, northern Bavaria, Germany; Middle Keuper Subgroup, Opole province, Poland

**Holotype:** BSPG 1931 X 502, partial skull

**Previously Referred Specimens:** None, although the phytosaur material from Krasiejów in Opole, southwest Poland, was considered highly similar to *Parasuchus angustifrons* by Butler *et al.* (2014), and one of these specimens, ZPAL AbIII 200, was noted to be ‘probably referable to *Parasuchus Angustifrons*’ by Kammerer *et al.* (2015).

**Specimen(s) Used for Scoring:** BSPG 1931 X 502; ZPAL AbIII 111; ZPAL AbIII 200

**Key References:** Kuhn (1936); Butler *et al.* (2014); Kammerer *et al.* (2015)

**Most Recent Diagnosis:** Butler *et al.* (2014) diagnosed *Parasuchus angustifrons* on the basis of the following proposed autapomorphies: 1) Stepped lateral rim of external naris that is strongly swollen and rugose at posterior end; 2) Paired depressions on the anterior portions of the nasals (immediately posterior to the external nares) and anterior portions of the frontals; 3) Foramen in ectopterygoid enlarged and subcircular in outline; 4) Suborbital foramen elongate and boomerang-shaped; 5) Large postparietal foramen at junction of supraoccipital and parietal.

**Modified Diagnosis:** Species of *Parasuchus* with the following autapomorphies: 1) Paired depressions on the anterior portions of the nasals; 2) Large postparietal foramen at junction of supraoccipital and parietal.

**Comments:** Of the characters proposed by Butler et al. (2013) as autapomorphic for *Parasuchus angustifrons*, characters 1 and 3 are both present in other members of *Parasuchus*: the laterally stepped and posteriorly rugose nares are also present in *Parasuchus hislopi* (ISI R42), while the enlarged ectopterygoid foramen is visible in the holotype of *Parasuchus bransoni* (FMNH UC 632). Character 4, an elongate and boomerang-shaped suborbital foramen, may be variable within the species, given that it appears to be absent in ZPAL AbIII 200. However, the degree of variability of this character among basal phytosaurs is difficult to assess, given the paucity of well-preserved palates generally within the genus *Parasuchus*. Given this uncertainty, character 4 should not be considered diagnostic until sufficient comparative material is available to assess its usefulness.

**‘*Paleorhinus*’ *parvus*** Mehl, 1928

**Age:** late Carnian–early Norian (*c.* 232–225 Mya)

**Occurrences:** Popo Agie Formation, Fremont County, Wyoming, USA

**Holotype:** MU 530, rostral/mandibular fragments and partial postcrania

**Previously Referred Specimens:** None

**Specimen(s) Used for Scoring:** MU 530

**Key References:** Mehl (1928); Kammerer *et al.* (2015)

**Most Recent Diagnosis:** Mehl (1928) did not provide a list of distinct autapomorphies; however, the morphology of ‘*Paleorhinus*’ *parvus* was discussed in relation to other phytosaurs, and a number of distinctive features were highlighted, which are discussed below.

**Modified Diagnosis:** Diagnosable on the basis of a unique combination of characters: 1) Anterior tip of the rostrum abruptly downturned; 2) Steep anterodorsally concave curve to the nares; 3) Anterior border of nares anterior to the antorbital fenestra; 4) Subnarial facial portion of the maxilla is dorsoventrally extensive.

**Comments:** ‘*Paleorhinus*’ *parvus* was interpreted by Mehl (1928) to possess a proportionately shorter prenarial rostrum than that of *Parasuchus bransoni*. The postnarial portion of the skull of ‘*Paleorhinus*’ *parvus* is not preserved; therefore, Mehl based his calculations on a partial mandible associated with the cranial remains, which he used to estimate total skull length. Mehl’s measurements suggest that the snout constituted 42.4% of total cranial length, which is indeed proportionately short compared to *Parasuchus bransoni*, for which values are approximately 50% (TMM 31100-101, 31100-418). This character should be treated with caution, however, as Mehl’s calculations of skull length based on the mandible may be somewhat inaccurate. Mehl also suggested that the rostrum of ‘*Paleorhinus*’ *parvus* is more slender than that of *Parasuchus bransoni*; however, it is uncertain whether this is due to mediolateral compression of the former.

The anterior tip of the rostrum was suggested by Mehl (1928) suggested to be more abruptly downturned than in *Parasuchus bransoni*. We concur with this assessment: the rostrum of ‘*Paleorhinus*’ *parvus* is more abruptly downturned than all species of *Parasuchus*, ‘*Paleorhinus*’ *sawini* and *Ebrachosuchus neukami,* and more closely approximates the rostral morphology of *Brachysuchus* and *Angistorhinus*. Mehl also noted that the nares of ‘*Paleorhinus*’ *parvus* are more steeply curved anterodorsally than *Parasuchus bransoni*, but considered the narial development to be suspect and ‘not dependable’. However, we concur with Kammerer *et al.* (2015) that there is no reason to suspect that the morphology of the narial region is not genuine, even considering lateral compression. Kammerer *et al.* (2015) also noted that the ‘subnarial facial portion of the maxilla’ is well preserved and ‘has greater relative height than that of *Parasuchus hislopi*’. We agree with this assessment, and find that this is also true when compared with all other members of *Parasuchus.*

**‘*Paleorhinus*’ *sawini*** Long & Murry, 1995

**Age:** late Carnian–early Norian (*c.* 232–225 Mya)

**Occurrences:** ‘Pre-Tecovas Horizon’, Dockum Group, Borden County, Texas, USA

**Holotype:** TMM 31213-16, skull and possibly associated isolated postcrania

**Previously Referred Specimens:** None

**Specimen(s) Used for Scoring:** TMM 31213-16

**Key References:** Long & Murry (1995); Stocker (2010)

**Most Recent Diagnosis:** Long & Murry (1995) diagnosed ‘*Paleorhinus*’ *sawini* on the basis of the following combination of characters: 1) Skull high; 2) Rostrum short and robust; 3) Prenarial length less than postnarial length; 4) Maxilla with strongly convex ventral margin; 5) Posterior portion of maxilla with prominent lateral flare; 6) Probable heterodont dentition; 7) Posterior maxillary alveoli enlarged and transversely rectangular; 8) Posterior premaxillary enlarged; 9) Upper dentition with 38 teeth.

**Modified Diagnosis:** A non-leptosuchomorph phytosaur with the following unique character complex: 1) Rostrum short and robust; 2) Prenarial length less than postnarial length; 3) Posterior premaxillary alveoli enlarged; 4) Upper dentition with 38 teeth; 5) Parietal/squamosal bars are medially bowed.

**Comments:** Characters 1, 4 and 5 of Long & Murry (1995) may be related to general large size and robusticity, but this has yet to be tested through ontogenetic studies of early phytosaurs. In particular, the height of the skull in proportion to its width does not appear to differ considerably from other species of early phytosaur such as *Parasuchus bransoni* (TMM 31100-8, 31100-101) or *Parasuchus angustifrons* (ZPAL Ab III 111, Ab III 200). Characters 6 and 7 are based on incomplete or missing morphology. No teeth remain in the skull, thus estimations of heterodonty are based solely on the shapes and relative sizes of empty alveolae. Given that some degree of heterodonty is present in all phytosaurs and the roots of even mediolaterally compressed phytosaur teeth are approximately circular, it is difficult to make meaningful statements about heterodonty without preservation of the actual dentition. Similarly, the posterior maxillary alveolae are heavily reconstructed with plaster, making character 7 questionable. It is unclear how intraspecifically variable tooth counts are in phytosaurs; however, specimens of *Parasuchus bransoni* and *Parasuchus hislopi* consistently have more than 40 teeth in the upper jaw (TMM 31100-101, 31100-239, ISI R42), whereas *Parasuchus angustifrons* appears to possess a similar number to ‘*Paleorhinus*’ *sawini* (ZPAL Ab III 200). This character is therefore retained, as further study may reveal it to contain taxonomic value.

Stocker (2010) noted two further characters that she proposed distinguish ‘*Paleorhinus*’ *sawini* from *Parasuchus*: 1) Shares medially-bowed parietal-squamosal bars with *Angistorhinus*; 2) The antorbital fossa is highly reduced or absent. It is, however, debatable how reduced in size the antorbital fossa was; upon close examination it appears possible that the fossa may have been broken up to its outer rim, i.e. to the extent of the concavity of the fossa. There is evidence of a thin lamina extending from the interior edge of the purported fenestra in TMM 31213-16, but all of its edges are broken away; this may be the remnants of the original antorbital fossa.

***Ebrachosuchus neukami*** Kuhn, 1936

**Age:** late Carnian (*c.* 232–228 Mya)

**Occurrences:** Blasensandstein (lateral equivalent of Hassberge Formation), Middle Keuper Subgroup, northern Bavaria, Germany

**Holotype:** BSPG 1931 X 501

**Previously Referred Specimens:** None

**Specimen(s) Used for Scoring:** BSPG 1931 X 501

**Key References:** Kuhn (1936); Long & Murry (1995); Butler *et al*. (2014)

**Most Recent Diagnosis:** Butler *et al.* (2014) diagnosed *E. neukami* based on six autapomorphies: 1) Preorbital length more than 3.8 times that of the orbit + postorbital length; 2) More than 50 teeth in the premaxilla and maxilla combined; 3) Pronounced, sharp flange extending along the lateral surface of the dorsal (postorbital) process of the jugal and the ventral (jugal) process of the postorbital that is continuous posteriorly with the lateral margin of the postorbital-squamosal bar; 4) Infratemporal fenestra is substantially longer anteroposteriorly than deep dorsoventrally, terminates anteriorly beneath the midpoint of the orbit; 5) Quadrate foramen very large, approximately two-thirds of width of foramen magnum; 6) Alveolar ridges absent from the anterior maxilla and only poorly developed on the premaxilla.

***Rutiodon carolinensis*** Emmons, 1856

**Age:** early Norian (*c.* 228–218 Mya)

**Occurrences:** Cumnock Formation, Deep River Coal Field, near Gulf, North Carolina, USA; Cumnock Formation, New Egypt coal mine, Chatham County, North Carolina, USA

**Neotype:** USNM PAL 214513 (formerly ‘Williams College unnumbered specimen’)

**Previously Referred Specimens:** Emmons’ original material (isolated, assorted postcrania, the location and accession numbers of which are unknown); 17 vertebrae and associated ribs (holotype of ‘*Clepsysaurus leai*’, location and accession numbers unknown); teeth, referred to various taxa by Emmons (1856) (location and accession numbers unknown); USNM V 5373 (holotype of ‘*Rhytidodon rostratus*’ Marsh, 1896); AMNH FR 1 (semi-complete skull plus composite skeleton); AMNH FR 2 (skull roof); AMNH FR 3 (right posterior portion of skull roof including squamosal and supratemporal fenestra); AMNH FR 4 (rostral fragment including partial nares and partial antorbital fenestra); AMNH FR 5 (Skull roof including left supratemporal region, quadrate and quadratojugal) (Gregory, 1962).

**Specimen(s) Used for Scoring:** USNM PAL 214513; USNM V 5373; AMNH FR 1–5

**Key References:** Emmons (1856; 1860); Marsh (1896); McGregor (1906); Colbert (1947); Gregory (1962); Hunt & Lucas (1989); Stocker (2010)

**Most Recent Diagnosis:** Stocker (2010) suggested the following diagnostic characters with reference to the neotype specimen: 1) Slender rounded premaxillae that lack a rostral crest; 2) Nares placed posteriorly between the antorbital fenestrae with borders above the level of the skull roof. To enable distinction between *Rutiodon carolinensis* and *Angistorhinus*, Stocker added a further character based on preserved temporal regions referred to *Rutiodon carolinensis* in the AMNH collections (AMNH FR 1–5): 3) Semi-depressed parietal-squamosal bars.

**Comments:** Hunt & Lucas (1989) designated a neotype for *Rutiodon carolinensis* and proposed four diagnostic characters that were centred around the temporal region of the skull. Stocker (2010) noted that none of the characters included in the diagnosis of Hunt & Lucas (1989) were observable in their designated neotype due to the temporal region not being preserved. Instead, the diagnostic appear to be based on USNM and AMNH material that was referred to *Rutiodon carolinensis* by Gregory (1962).

Characters 1 and 2 of the diagnosis of Stocker (2010) are observable in the neotype; however, if only these characters are considered it is not possible to differentiate *Rutiodon carolinensis* from *Angistorhinus*. Stocker also therefore used the referred AMNH material to provide a further character and a more robust diagnosis.

It should be noted that the elevation of the nares (character 2 of Stocker, 2010) may not be entirely reliable, as the orbital and narial portions of the neotype do not articulate together meaning there is no way to be certain that the nares would have been elevated above the level of the skull roof. Furthermore, Gregory (1962) noted that in the neotype the nares were inferred to be elevated, while those of AMNH FR 1 are not, suggesting a degree of variability in this feature*.*

***Angistorhinus talainti*** Dutuit, 1977

**Age:** late Carnian–early Norian (*c.* 232–225 Mya)

**Occurrences:** Timezgadiouine Formation, Western Moroccan Atlas, Morocco

**Holotype:** MNHN TAL 1–11 (syntypes), three skulls plus four partial mandibles and mandibular fragments

**Previously Referred Specimens:** None

**Specimen(s) Used for Scoring:** MNHN TAL 1; MNHN TAL 2

**Key References:** Mehl (1913); Dutuit (1977); Long & Murry (1995)

**Most Recent Diagnosis:** Long & Murry (1995) felt the placement of *A. talainti* within the genus *Angistorhinus* was unjustified and therefore considered it as referable to a new and currently unnamed genus within the subfamily ‘Angistorhininae’ Camp, 1930. They considered it to be diagnosed by the following combination of characters: 1) Supratemporal fenestra extremely elongate and narrow, crescentic in dorsal view and extending to posterolateral corner of squamosal; 2) Postorbital portion of the skull elongate with parietals long, narrow and fused for most of their length; 3) Postfrontal not in contact with supratemporal fenestra; 4) Occiput with shallow posterior emargination; 5) Posterior process of squamosal well developed and terminally convex; 6) Parietal extensions not present; 7) Opisthotic process short and paddle shaped.

**Modified Diagnosis:** *A. talainti* can be diagnosed from other phytosaurs using a combination of characters that includes generic *Angistorhinus* characters and characters recognized in previous studies (Dutuit, 1977; Long & Murry, 1995): 1) Parietal/squamosal bars equal to, or greater than the thickness of the postorbital/squamosal bars; 2) Parietal/squamosal bars curve medially; 3) Supratemporal fenestrae narrow and short; 4) Postorbital portion of the skull elongate with parietals long, narrow and fused for most of their length; 5) Posterior process of squamosal well developed and terminally convex; 6) Posterolaterally curving groove extends from the supratemporal fenestra to the posterolateral corner of the squamosal; 7) Prominent ridge runs along the anterior dorsolateral edge of the postorbital-squamosal bar; 8) Parietals diverge posterior to the main vacuity of the supratemporal fenestra.

**Comments:** The interpretation of the supratemporal fenestra of *A. talainti* in character 1 of Long & Murry (1995) contradicts the description of Dutuit (1977), who stated in his diagnosis that the supratemporal fenestrae are narrow and short. The figures in Dutuit (1977) superficially show an elongate fenestra, as suggested by Long & Murry (1995); however, Long & Murry did not distinguish between the dorsal opening of the fenestra and a posterior groove which grades from the posterior corner of the fenestra onto the posterolateral corner of the squamosal. It is difficult to delimit the fenestra due to the presence of this groove, but we agree with Dutuit (1977), that the actual opening of the fenestra is relatively short. Characters 3 and 4 of Long & Murry (1995) appear to be shared by all members of *Angistorhinus* observed in this study, and character 5 is present in multiple specimens from Texas and New Mexico, such as TMM 31100-1332, TMM 31100-164, TMM 31100-298 and NMMNHS P-4781. Character 6 is difficult to interpret - the terms ‘parietal extensions’ and ‘horizontal parietal extensions’ are used in Long & Murry’s revised diagnosis of ‘Angistorhininae’; however, no further explanation of these features is given. We assume that this terminology refers to the posterior thickening of the parietal/squamosal bars that is observed throughout *Angistorhinus*. The parietals of *A. talainti* are expanded posteriorly in a horizontal plane to a greater extent than other species and specimens of *Angistorhinus* mentioned by Long & Murry (1995).

***Angistorhinus grandis*** Mehl, 1913

**Age:** late Carnian–early Norian (*c.* 232–225 Mya)

**Occurrences:** Popo Agie Formation, between Squaw and Baldwin Creeks, Fremont County, Wyoming, USA

**Holotype:** FMNH UC 631, Skull and associated partial mandible

**Previously Referred Specimens:** None

**Specimen(s) Used for Scoring:** FMNH UC 631

**Key References:** Mehl (1913); Long & Murry (1995)

**Most Recent Diagnosis:** Long & Murry (1995) diagnosed *A. grandis* as a species of *Angistorhinus* with the following characters: 1) Posterior process of squamosal very short, but deep with straight posterior margin; 2) No cleft between posterior process and descending process of the squamosal; 3) Parietal extension ?short; 4) Rostrum delicate; 5) Alveoli circular throughout with posterior premaxillary alveoli not enlarged (this region of snout is not swollen); 6) Ventral margin of maxilla not laterally flared; 7) Orbits directed more dorsally than laterally.

**Modified Diagnosis:** We diagnose *A. grandis* as a species of *Angistorhinus* with the following unique character combination: 1) Ventral margin of maxilla not laterally flared; 2) Supratemporal fenestrae wide and triangular in shape; 3) Lateral temporal fenestra large and more sub-triangular than rectangular; 4) U-shaped emargination between the supratemporal fenestrae at the posterior border of the parietals in dorsal view.

**Comments:** We suggest that characters 1 and 2 of Long & Murry (1995) should be treated with caution, or potentially rejected, as the posterior process of the squamosal is not actually preserved in *A. grandis*. The morphology of the posterior process was reconstructed in plaster from a supposed impression of the medial surface of the process preserved in the matrix with the skull; however, no photographs or diagrams exist of this impression, and its described morphology differs from the rounded morphology of the posterior process of the squamosal observed in all other specimens of *Angistorhinus.*

Characters 3 and 4 of Long & Murry (1995) are phrased ambiguously and are therefore difficult to objectively assess. Alongside character 5, these characters are also present in all other specimens of *Angistorhinus* that were examined, and are therefore not useful for a diagnosis at specific level. Character 7 reports the orientation of the orbit; however, phytosaur skulls are often mediolaterally or dorsoventrally compressed meaning that the orientation of the orbits can vary widely both inter- and intraspecifically, and should not be used for diagnostic or phylogenetic purposes (Chatterjee, 1978; Hungerbühler, 1998).

***Smilosuchus gregorii*** (Camp, 1930)

**Age:** early Norian (*c.* 225–220 Mya)

**Occurrences:** Blue Mesa Member, Chinle Formation, near Round Rock, Apache County, Arizona, USA

**Holotype:** UCMP 27200, slightly dorsoventrally compressed skull and mandibles

**Previously Referred Specimens:** UCMP A270/27192; UCMP A270/27195 (Camp, 1930); AMNH FR 3060 (Colbert, 1947); USNM V 18313; UCMP 63921; UCMP 35737; UMMP 14366; PPHM WT 3217; PPHM WT 3214; PPHM WT 3230 (Long & Murry, 1995).

**Specimen(s) Used for Scoring:** UCMP 27200; AMNH FR 3060

**Key References:** Camp (1930); Colbert (1947); Long & Murry (1995)

**Most recent diagnosis:** Long & Murry (1995) diagnosed the new genus *Smilosuchus* and the sole species that they referred to it, *S. gregorii*, based on the following character combination: 1) Extreme heterodonty; 2) Posterior premaxillary teeth (except last three) abruptly and very greatly enlarged, causing a swelling of the premaxilla in this region; 3) Tooth pattern posteriorly shifted; 4) Ventral margin of the maxilla greatly flared laterally; 5) Rostral crest fully developed; 6) Anterior portion of rostrum very heavy and massive; 7) Posterior portion of the skull considerably wider than in *Leptosuchus*, with lateral temporal fenestra facing dorsolaterally; 8) Orbit directed dorsolaterally.

**Modified Diagnosis:** *S. gregorii* is diagnosed on the basis of the following unique character combination: 1) Full rostral crest; 2) Greatly dorsoventrally expanded posterior process of the squamosal.

**Comments:** Characters 4, 6 and 7 of Long & Murry (1995) may be size-correlated, which is both undesirable and problematic (Irmis, 2005); 7 and 8 are highly prone to taphonomic distortion and the phrasing of character three is ambiguous.

Of the remaining characters, 2 and 5 are consistently present only in *S. gregorii* rather than ‘*S*’. *adamanensis* or ‘*S*’. *lithodendrorum* and may therefore be useful in defining the species. Character 1 (extreme heterodonty) is present in all current species of *Smilosuchus* and is therefore not of diagnostic use for *S. gregorii*.

**‘*Smilosuchus*’ *adamanensis*** (Camp, 1930)

**Age:** early Norian (*c.* 225–220 Mya)

**Occurrences:** Blue Mesa and lower Sonsela Members, Chinle Formation, Petrified Forest National Park, Apache County, Arizona, USA

**Holotype:** UCMP 26699, skull and mandibles

**Previously Referred Specimens:** All phytosaur material from UCMP/PEFO localities PFV 122, PFV 120, PFV 121, PFV 142, PFV 157 and PFV 155 (Camp, 1930; Parker, 2002); (UCMP 26696, 26697, 26698, 26706, 26717, 26718, 26720, 26725, 26727, 26729, 26730, 26731, 26756, 27070, 27093, 27094, 27104 and 27106 referred as paratypes (Camp, 1930)); UCMP 27099, 27006, 27007, 27008, 27010, 27011, 27013, 27014, 27015, 27025, 27026, 27027 (Camp, 1930); UCMP 27446; MNA V3024; MNA V3025; MNA V2675; MNA V3698; UCMP 27444; UCMP 27185; UCMP 27036 (holotype of ‘*M*’. *zunii*); UCMP 27060; UCMP 126991; USNM V 15841; UCMP 124957; USNM (NPS 72-39 in part); AMNH (EHC 1946-23); UCMP 26688 (holotype of ‘*S*’. *lithodendrorum*); UMMP 7523 (holotype of ‘*Leptosuchus imperfecta*’) (Long & Murry, 1995). PEFO 34852 (Griffin et al., 2017).

**Specimen(s) Used for Scoring:** UCMP 26699; UCMP 170166

**Key References:** Camp (1930); Long & Murry (1995); Stocker (2010)

**Most Recent Diagnosis:** Long & Murry (1995) differentiated ‘*S’. adamanensis* from other *Leptosuchus*-grade phytosaurs on the basis the following character combination: 1) Posterior process of squamosal is a deep vertical plate with moderate posterior elongation beyond paroccipital process; 2) Post-fenestral portion of squamosal wide and abruptly truncated when viewed dorsally, though the extra-fenestral portion of the bar is narrow.

**Comments:** Both characters of Long & Murry are accurate; however, character 1 is somewhat variable in other early leptosuchomorph taxa e.g., *Leptosuchus crosbiensis* (UMMP 7522, TMM 31173-120), and character 2 is based on heavily distorted morphology in the holotype. The lack of either a rostral or narial crest in ‘*S.*’ *adamanensis* distinguishes it from other putative members of the genus *Smilosuchus* and members of *Leptosuchus*, though it is unclear whether or not this feature is plesiomorphic.

‘*S.*’ *adamanensis* suffers the same problem as ‘*S*’. *lithodendrorum* (see below); the majority of material referred to this species by Camp (1930) was referred based on geographical and stratigraphical proximity. As such, previous definitions of the taxon may be chimeric; subsequent analyses should therefore treat referred specimens with caution or rely only on the holotype. A thorough re-examination and redescription of the holotype of ‘*S’. adamanensis* may bring further diagnostic characters to light; however, such work is beyond the scope of the current study.

**‘*Smilosuchus*’ *lithodendrorum*** (Camp, 1930)

**Age:** Norian (*c.* 219–217 Mya)

**Occurrences:** Lot’s Wife beds, Sonsela Member, Chinle Formation, Petrified Forest National Park, Navajo County, Arizona, USA; Tecovas Formation, Dockum Group, Crosby County, Texas, USA

**Holotype:** UCMP 26688, poorly preserved, fragmentary and compressed left half of skull, and almost complete mandibles.

**Previously Referred Specimens:** All phytosaur material from UCMP/PEFO localities PFV 108, PFV 096, PFV 172, PFV 146 and PFV 161 (Camp, 1930; Parker, 2002); (UCMP 26683, 26684, 26719, 27179, 27189, 27181 and 27182 referred as paratypes (Camp, 1930)); UCMP 27151, 26693, 26694, 27017, 27183, 27184, 27149 (Camp, 1930); TMM 31173-121 (Stocker, 2010).

**Specimen(s) Used for Scoring:** UCMP 26688; TMM 31173-121

**Key References:** Camp (1930); Long & Murry (1995); Stocker (2010); Parker & Martz (2011)

**Most Recent Diagnosis:** Stocker (2010) proposed that ‘*Smilosuchus*’ *lithodendrorum* should be diagnosed as a species of *Smilosuchus* with the following characters: 1) A highly angled rostrum that continues anteroventrally in a smooth descent; 2) The posterior process of the squamosals grade anteroventrally into the opisthotic process; 3) Very slight medial flange on the dorsal edge of the squamosal.

**Comments:** The validity of ‘*S*’. *lithodendrorum* has previously been questioned; it was synonymized with *Leptosuchus crosbiensis* by Long & Murry (1995) with no justification given. Stocker (2010) suggested that this may have been due to the narrow postorbital/squamosal bar, which is a prominent feature of *Leptosuchus crosbiensis* and *Leptosuchus studeri.* It is also possible that the synonymization was due to extensive morphological variation exhibited in the specimens referred to ‘*S*’. *lithodendrorum* by Camp (1930). Camp’s assignments of lectotypes and referred specimens were based on geographical and stratigraphical proximity of specimens to the holotype, rather than morphology; as a result, a number of smaller, non-crested skulls (between 678 and 965 mm in length) were assigned to this species as juveniles of ‘*S.*’ *lithodendrorum* (UCMP 26684, 26719, 27179, 27181). These specimens lack the full rostral crest reported in larger individuals of ‘*S.*’ *lithodendrorum*, instead their crests extend anterior of the nares before descending to form tubular rostra close to the most anterior extent of the maxillae. This morphology, combined with the size range of the specimens and aforementioned similarities between *L. crosbiensis* and ‘*S.*’ *lithodendrorum*, seemingly makes them indistinguishable from the holotype of *L. crosbiensis.* Conversely, the crest morphology in larger specimens of both ‘*S.*’ *lithodendrorum* and *L. crosbiensis* does differ substantially. In larger specimens of ‘*S*’*. lithodendrorum* (e.g., UCMP 26688; TMM 31173-121) the rostrum is fully crested, with the crest forming a straight diagonal gradient from the nares to the tip of the premaxillae. However, in larger specimens of *L. crosbiensis* (e.g., TMM 43684, 43684-8) the morphology remains unchanged from smaller specimens such as TMM 31173-120, with a partial rostral crest extending from the nares to the most anterior extent of the maxillae, and a separate premaxillary crest at approximately the mid-point of the premaxillae. A more detailed examination of all material potentially referable to these taxa may help to quantify the morphological variation associated with these taxa, and disentangle their diagnoses. Stocker (2010) did tentatively identify some subtle differences between ‘*S.*’ *lithodendrorum* and *L. crosbiensis*, although her diagnosis does not take into account the intermediate morphologies present in some specimens. However, a full redescription and re-evaluation of the species is beyond the remit of this paper. We therefore use the diagnosis of Stocker (2010).

**‘*Phytosaurus*’ *doughty*** Case, 1920

**Age:** early Norian (*c.* 225–220 Mya)

**Occurrences:** Tecovas Formation, Dockum Group, Texas, USA

**Holotype:** AMNH FR 4919, right posterior portion of skull

**Previously Referred Specimens:** Possibly MSM 92-023.001 (Stocker, 2010).

**Specimen(s) Used for Scoring:** AMNH FR 4919

**Key References:** Case (1920); Stocker (2010)

**Most Recent Diagnosis:** Case (1920) was able to identify two characters to separate ‘*Phytosaurus*’ *doughtyi* from other *Leptosuchus*-grade phytosaurs: 1) No evidence of the opisthotic process posterior to the quadrate; 2) The nares rise at their posterior rim.

**Comments:** Character 2 of Case (1920) is present in referred specimens of *Leptosuchus crosbiensis* (TMM 31173-120 and TTU-P 09230); despite this, Stocker (2010) tentatively suggested the referral of MSM 92-023.001 to ‘*Phytosaurus*’ *doughtyi* based in part on the presence of a deep saddle between the orbits and external nares. It may therefore be the case that the differences in this character are more nuanced between *L. crosbiensis* and ‘*Phytosaurus*’ *doughtyi*, rather than being simply present or absent. Character 1 of Case (1920) appears to be an autapomorphy of ‘*Phytosaurus*’ *doughtyi*, and this is supported by our first-hand study of the holotype specimen.

***Leptosuchus studeri*** Case & White, 1934

**Age:** early Norian (*c.* 225–220 Mya)

**Occurrences:** Tecovas Formation, Dockum Group, Cerita de la Cruz Creek, Potter County, Texas USA; Blue Mesa Member, Chinle Formation, Arizona, USA

**Holotype:** UMMP 14267, skull

**Previously Referred Specimens:** None

**Specimen(s) Used for Scoring:** UMMP 14267

**Key References:** Case & White (1934); Stocker (2010)

**Most Recent Diagnosis:** Stocker (2010) used two characters to diagnose *L. studeri*: 1) Posterior edges of the posterior processes [of the squamosals] curl inwards in *L. studeri*, differing from the straight posterior processes of *L. crosbiensis*; 2) Small dorsally convex area on the dorsal surface of the mid-premaxillae.

**Comments:** At the time of writing the holotype and referred specimens of *L. studeri* were unavailable to study, so our observations are tentative and brief. It seems possible that character 1 of Stocker (2010) could be due to taphonomic distortion, although the morphology is present on both posterior processes, and to the same degree. Character 2 of Stocker (2010) is also present in *Leptosuchus crosbiensis*, appearing in a line-drawing of the holotype (Case, 1922), and is visible in multiple other specimens (TMM 31173-120, 43684, 43684-8; TTU-P 00902, 09230, 09234, 10001). The shape of the premaxillary crest does, however, appear to be different to that of *L. crosbiensis*. The crest of *L. crosbiensis* is anteroposteriorly symmetrical and forms a smooth ‘hump’, whereas in *L. studeri* the anterior portion of the crest slopes gently and the posterior slightly more steeply; the apex of the crest is therefore sharper. A partially prepared specimen at Petrified Forest National Park (field no. RLG 11/07-3) also displays this crest morphology, and the posterior process of the squamosal is also identical in lateral view to *L. studeri*.

***Leptosuchus crosbiensis*** Case, 1922

**Age:** early Norian (*c.* 225–220 Mya)

**Occurrences:** Tecovas Formation, Dockum Group, Crosby County, Texas, USA

**Holotype:** UMMP 7522, skull

**Previously Referred Specimens:** USNM V 15481 (Stocker, 2010); PPHM WT 3243; UCMP 27179; UCMP 27181; UCMP 26688 (Holotype of ‘*S.* *lithodendrorum*’); UCMP 126992; UCMP 126988; UCMP 126745; UCMP 27195; UCMP 27192; MSM 92-023.001; UMMP 14267 (Holotype of *L. studeri*); UMMP 14366; UMMP 12198; TTU-P ‘383’; TMM 1010-5; TMM 31173-120; TMM 31173-121 (Long & Murry, 1995).

**Specimen(s) Used for Scoring:** UMMP 7522; TMM 31173-120; USNM V 15841; TTU-P 09230

**Key References:** Case (1922); Long & Murry (1995); Stocker (2010)

**Most Recent Diagnosis:** Stocker (2010) listed diagnostic features of *L. crosbiensis* in comparison to other *Leptosuchus*-grade taxa: 1) An overall slenderness of the skull; 2) Supratemporal fenestrae that are completely visible in dorsal view; 3) Supratemporal fenestrae are bounded anterolaterally by mediolaterally narrow dorsal edges of the squamosals.

**Modified Diagnosis:** 1) An overall slenderness of the skull; 2) Supratemporal fenestrae that are completely visible in dorsal view; 3) Supratemporal fenestrae are bounded anterolaterally by mediolaterally narrow dorsal edges of the squamosals; 4) Dorsally rounded crest on the anterior portion of the premaxilla; 5) Partial rostral crest extends approximately level from the nares and descends at a point level with the anterior-most extent of the maxillae.

**Comments:** We suggest two additional characters (4 and 5 in the above modified diagnosis), to reinforce the diagnosis of *L. crosbiensis*. See ‘*Smilosuchus*’ *lithodendrorum* for further discussion of this taxon*.*

***Pravusuchus hortus*** Stocker, 2010

**Age:** Norian (*c.* 219–217 Mya)

**Occurrences:** White ‘hoodoo’ sandstone, Kellogg Butte Sandstone, Sonsela Member, Chinle Formation, Devil’s Playground, Petrified Forest National Park, Arizona, USA; ?Monitor Butte Member, Chinle Formation, Fry Canyon, Utah, USA

**Holotype:** AMNH FR 30646, dorsoventrally crushed postnarial portion of skull and separate mediolaterally compressed right half of rostrum

**Previously Referred Specimens:** PEFO 31218; PEFO 34239 (Stocker, 2010); UMNH 28293 (McCormack & Parker, 2017).

**Specimen(s) Used for Scoring:** AMNH FR 30646; PEFO 31218; PEFO 34239

**Key References:** Stocker (2010); Parker & Martz (2011); McCormack & Parker (2017)

**Most Recent Diagnosis:** Stocker (2010) identified one autapomorphy for *Pravusuchus hortus* (the ‘septomaxilla’ forms part of the lateral rim of the external nares) and a unique character combination: 1) Absence of antorbital fossa; 2) broad and rounded interpremaxillary fossa; 3) Alveolar ridges visible in lateral view; 4) Fully crested rostrum; 5) Long posterior process of squamosal; 6) Posterior process of squamosal is greatly dorsoventrally expanded; 7) Possession of a subsidiary opisthotic process of the squamosal; 8) Supratemporal fenestrae partially depressed; 9) Supratemporal fenestrae that are mostly visible in dorsal view.

**Comments:** In relation to the ‘septomaxillary’ autapomorphy of *Pravusuchus hortus* Stocker (2010) stated that ‘dorsal examination of the narial region shows a possible dorsolateral process of the ‘septomaxilla’ on the lateral border of the naris’. Upon first-hand examination of the holotype and other specimens we suggest that a lateral extension of the ‘septomaxilla’ is unlikely; rather, the suture identified by Stocker may represent the lateral border of the paranasal, as described by Hungerbühler *et al*. (2013). As noted in the holotype by Stocker (2010), ‘iron oxide covers potential ‘septomaxilla’-premaxilla sutures’; however, amongst the iron oxide a distinct groove extends from the anterior narial border, occupying the same position as the ‘septomaxillary’ suture in most phytosaurs. Therefore, we suggest that *Pravusuchus hortus* is diagnosed using only the unique character combination proposed by Stocker (2010), until the lateral extent of the ‘septomaxillae’ can be unambiguously verified in additional specimens.

***Nicrosaurus kapffi*** (Meyer, 1860)

**Age:** middle–late Norian (*c.* 216–209 Mya)

**Occurrences:** Löwenstein Formation (middle Stubensandstein), Middle Keuper Subgroup, Baden-Württemburg, Germany

**Holotype:** SMNS 4060/4060a, poorly preserved rostral fragment and associated symphyseal-postsymphyseal mandibular fragment, uncat. No. 15, left maxillary and jugal fragment that fits with 4060, SMNS 54708, anterior fragment of left premaxilla (syntype series)

**Previously Referred Specimens:** SMNS 4378; SMNS 4379; SMNS 5725; SMNS 5726, SMNS 5727; SMNS 13078; SMNS 54706; SMNS 56989; SMNS uncat. No. 12; SMNS 4380; SMNS 5730; SMNS uncat. No. 9; SMNS 54708; NHMUK 38036; NHMUK 38043; NHMUK 42743; NHMUK 42744; GPIT 2223.000; GPIT uncat. No. 399 (Hungerbühler, 1998).

**Specimen(s) Used for Scoring:** SMNS 4378; SMNS 4379; SMNS 5726; SMNS 5727; NHMUK 42743

**Key References:** Meyer (1860, 1861, 1863, 1865); Hungerbühler (1998); Hungerbühler & Hunt (2000)

**Most Recent Diagnosis:** Hungerbühler (1998) presented a unique character combination for *N. kapffi* based largely on characters from Long & Murry (1995) and Ballew (1989). The unique combination is composed of the following two characters: 1) Presence of a continuous prenarial crest reaching just behind the downturned tip of the snout; 2) Top of prenarial crest straight or slightly convex.

***Nicrosaurus meyeri*** Hungerbühler & Hunt (2000)

**Age:** middle–late Norian (*c.* 216–209 Mya)

**Occurrences:** Löwenstein Formation (middle Stubensandstein), Middle Keuper Subgroup, Baden-Württemburg, Germany

**Holotype:** SMNS 12593, dorsoventrally compressed skull in two pieces

**Previously Referred Specimens:** SMNS 4059, 12593/2 & SMNS uncat. No. 11; NHMUK 38038 & 42745; GPIT 261/001 & GPIT 2070.001 (Hungerbühler & Hunt, 2000).

**Specimen(s) Used for Scoring:** SMNS 12593; SMNS 4059; NHMUK 38038; NHMUK 42745; GPIT 2070.001

**Key References:** Meyer (1861); Hunt (1994a); Hungerbühler (1998); Hungerbühler & Hunt (2000)

**Most Recent Diagnosis:** Hungerbühler & Hunt (2000) identified *N. meyeri* as a species of *Nicrosaurus*, differentiated from *N. kapffi* on the basis of the following characters: 1) Rostrum slender and gracile; 2) Prenarial crest absent or over posterior part of the snout only; 3) Septomaxilla terminates at the level of the anterior tip of the nasal; 4) Anterior part of the internasal septum may be prominent and visible in lateral view; 5) Sculpture of the pre-orbital region prominent; 6) Cheek region (quadratojugal + jugal) always with a well developed longitudinal depression; 7) Craniomandibular facet of the quadrate less wide and lateral condyle offset from the cheek; 8) Postorbito-squamosal bar broader; 9) Supratemporal fenestra less wide; 10) In dorsal view the posterior process of the squamosal is continuously broad, the medial rim is angular; 11) Posterior process of the squamosal terminates with a pointed tip; 12) Paroccipital process of the squamosal (‘hooklike process’) small; 13) Alveolar ridges more prominent, prechoanal part of the vomers mostly slender and tapering; 14) Upper jaw dentition moderately heterodont; 15) Premaxillary teeth set more laterally; 16) Mandibular symphysis equals ~50% of the total mandibular length (rather than 40% as in *Nicrosaurus kapffi*)*.*

***Coburgosuchus goeckeli*** Heller, 1954

**Age:** middle–late Norian (*c.* 216–209 Mya)

**Occurrences:** Upper Burgsandstein (lateral equivalent of the Löwenstein Formation), Untersiemau, Baden-Wurttemburg, Germany

**Holotype:** NMC 15436, postnarial portion of skull

**Previously Referred Specimens:** None

**Specimen(s) Used for Scoring:** NMC 15436

**Key References:** Heller (1954)

**Most Recent Diagnosis:** Heller (1954) originally described this specimen in German; however, since then this taxon has been largely ignored. Heller (1954) diagnosed the genus as a phytosaur of similar size to *Nicrosaurus kapffi*, though differentiated by an even greater development of the squamosals and more laterally oriented orbits.

**Comments:** As with many other diagnoses, we doubt the utility of orbital orientation as a reliable diagnostic character; however, the substantially more pronounced lateral curvature of the squamosals does appear to be valid and distinguishes *Coburgosuchus goeckeli* from *Nicrosaurus kapffi*. We are currently preparing a redescription of *Coburgosuchus*, and as such we do not present further diagnostic characters at this time.

**‘*Machaeroprosopus*’ *zunii*** Camp, 1930

**Age:** early Norian (*c.* 225–220 Mya)

**Occurrences:** Blue Mesa Member, Chinle Formation, Arizona, USA

**Holotype:** UCMP 27036, partial braincase and postcrania

**Previously Referred Specimens:** All specimens from UCMP localities 7307, 7308, 7309, 7310 & A 255; UCMP 27041, 27044, 27054, 27154, 27155, 27156, 27189, 27158, 27056, 27057, 27159, 27048 & 27189 (Camp, 1930).

**Specimen(s) Used for Scoring:** UCMP 27159

**Key References:** Camp (1930); Stocker (2010)

**Most Recent Diagnosis:** Camp (1930) produced a diagnosis of ‘*M*’. *zunii* based on various referred specimens. Due to the arbitrary referral of many of the specimens (see below) the characters presented may constitute a mosaic from multiple species: 1) Rostrum very long, slender; 2) Nasals large, extending forward beyond nares and entering dorsal border of antorbital fenestra; 3) Posterior squamosal process very large, broad, flat, and expanded vertically; 4) Parietals small, anterior suture lies well behind posterior border of orbits; 5) Posterior parietal process Y-shaped; 6) Anterior border of supratemporal fenestra very wide and not excavated forward to or nearly to middle of parietals; 7) Postorbito-squamosal bar narrow.

**Comments:** Camp (1930) presented the holotype of ‘*M*’. *zunii* as a partial braincase and postcrania, thus containing little to no material of diagnostic utility. However, a number of specimens containing cranial material, such as UCMP 27048, 27189, 27159 were also referred to ‘*M*’. *zunii*, of which UCMP 27159 was also used in the matrix of Stocker (2010). As the type material is undiagnostic the rest of the referred specimens are referred on the basis of stratigraphic and geographic proximity to the type and should therefore be treated cautiously or altogether excluded. Additionally, inspection of the holotype specimen suggests that the braincase belongs to a smaller individual than the postcrania, and likely belongs to a taxon less derived than basal leptosuchomorph phytosaurs, suggesting that the holotype specimen may also be chimaeric (Axel Hungerbühler, pers. comm. to ASJ, 2018). ‘*M*’. *zunii* is included here and scored based on only one of Camp’s referred specimens. A specimen in the Smithsonian Institute (USNM V17098) is catalogued as ‘*M*’. *zunii* and appears to share a similar morphology to that of the specimens referred by Camp; we include this specimen as a separate OTU to test the credibility of its referral and this taxon in general, to judge whether it warrants more detailed investigation.

***Protome batalaria*** Stocker, 2012

**Age:** early Norian (*c.* 220–218 Mya)

**Occurrences:** Upper Lot’s Wife beds, Sonsela Member, Chinle Formation, Petrified Forest National Park, Arizona, USA

**Holotype:** PEFO 34034, pre-narial rostrum with narial fragments, post-narial skull roof with squamosals, basioccipital and left mandible.

**Previously Referred Specimens:** None

**Specimen(s) Used for Scoring:** PEFO 34034

**Key References:** Stocker (2012)

**Most Recent Diagnosis:** Stocker (2012) presented three autapomorphies and a unique character combination for *Protome batalaria*. Due to the length of the unique character combination we only present the autapomorphies here: 1) Presence of a flat ventral surface on the basitubera; 2) Posterior prongs from the exoccipitals dorsal to the foramen magnum; 3) Fossa surrounding the anterior corner of the external mandibular fenestra.

**Modified Diagnosis:** The unique character combination of Stocker (2012), plus the two autapomorphies: 1) Presence of a flat ventral surface on the basitubera; 2) Fossa surrounding the anterior corner of the external mandibular fenestra.

**Comments:** Autapomorphies 1 and 3 of Stocker (2012) appear valid; however, the posterior exoccipital prongs mentioned in character 2 appear to also be present in *Coburgosuchus*, but further study is required to verify this. These prongs were also noticed by Hungerbühler *et al.* (2013) in *Machaeroprosopus lottorum* and were identified as the proatlantes; this feature is also present in multiple other specimens referable to *Machaeroprosopus*, ‘*Redondasaurus*’ and potentially other taxa, although a thorough review is required (Axel Hungerbühler, pers. comm. to ASJ, 2018).

**‘*Machaeroprosopus*’ *andersoni*** Mehl, 1922

**Age:** late Norian (*c.* 218–208 Mya)

**Occurrences:** Bull Canyon Formation, Dockum Group, near Santa Rosa, Guadalupe County, New Mexico, USA

**Holotype:** FMNH UC 396, heavily reconstructed skull

**Previously Referred Specimens:** None

**Specimen(s) Used for Scoring:** FMNH UC 396

**Key References:** Mehl (1922); Long & Murry (1995)

**Most Recent Diagnosis:** Mehl (1922) suggested that ‘*M*’. *andersoni* shares a close affinity with ‘*Machaeroprosopus validus*’ and distinguished it using the following characters: 1) Postero-median border of supra-temporal fenestrae not completely depressed; 2) Anterior border of nares not elevated; 3) Terminal expansion of rostrum gradual; 4) Nasals extending some distance in front of anterior border of nares; 5) Greatest length of maxillae at alveolar margin; 6) Approximately ninety-four teeth in upper dentition; 7) Four large teeth in terminal expansion of rostrum; 8) Alveolae not crowded; 9) Lateral expansion of rostrum at posterior end of premaxillae.

**Comments:** ‘*M*’. *andersoni* was synonymized with *Machaeroprosopus buceros* by Long & Murry (1995); however, upon inspection of their diagnosis of *Machaeroprosopus buceros* it is clear that many characters are inappropriate for, or are not preserved in the holotype (and only specimen) of ‘*M*’. *andersoni*. Two characters pertain to the squamosals, which are not preserved in ‘*M*’. *andersoni*, three characters are unnecessarily used to describe the same morphology of the pre-narial crest, and the majority of the remaining characters do not distinguish either taxon from many others, even when the characters are taken in combination.

Aside from the problem that the characters of Mehl (1922) may not differentiate ‘*M*’. *andersoni* from all current species of *Machaeroprosopus*, they are also based on comparison with ‘*Machaeroprosopus validus*’ which has subsequently been lost and also found not to be the type species of *Machaeroprosopus* (Parker *et al.*, 2012)*.* This taxon requires reanalysis and thorough comparison to *Machaeroprosopus buceros* (the valid type species) and other members of *Machaeroprosopus*; however, this is beyond the scope of the current study.

***Machaeroprosopus jablonskiae*** Parker & Irmis, 2006

**Age:** late Norian (*c.* 218–216 Mya)

**Occurrences:** Jim Camp Wash beds, Sonsela Member, Chinle Formation, Petrified Forest National Park, Arizona, USA

**Holotype:** PEFO 31207, skull roof with squamosals

**Previously Referred Specimens:** None

**Specimen(s) Used for Scoring:** PEFO 31207

**Key References:** Parker & Irmis (2006); Parker & Martz (2011)

**Most Recent Diagnosis:** Parker & Irmis (2006) noted a single autapomorphy of *Machaeroprosopus jablonskiae*: Distinct smooth bevelled edge on the antero-medial edge of the postorbito-squamosal bar that forms a supratemporal fossa lateral to the supratemporal fenestra. This feature has subsequently also been identified in *Machaeroprosopus lottorum* (Hungerbühler *et al.*, 2013); however, *Machaeroprosopus lottorum* is distinguishable as the bevelled edge effectively closes the supratemporal fenestra in dorsal view, whereas the fenestra remains visible in *Machaeroprosopus jablonskiae*. Alongside this they presented a unique character combination as follows: 1) Apomorphic characters for *Pseudopalatus* [*Machaeroprosopus*] clade; 2) Squamosal tips that are not knob-like as in *Machaeroprosopus buceros* + *Machaeroprosopus pristinus*; 3) Thin oar-like paroccipital process of the opisthotic that is fused to the internal squamosal process as in *Machaeroprosopus buceros* + *Machaeroprosopus mccauleyi*; 4) Anterior process of the squamosal enters the lateral wall of the braincase as in *Mystriosuchus westphali* and *S. gregorii*; 5) No lateral groove or ridge on the squamosal; 6) Squamosals are strongly anteroposteriorly shortened*.*

***Machaeroprosopus mccauleyi*** (Ballew, 1989)

**Age:** late Norian–early Rhaetian (*c.* 213–207 Mya)

**Occurrences:** Upper Petrified Forest Member, Chinle Formation, Billings Gap, Apache County, Arizona, USA

**Holotype:** UCMP 126999, skull missing anterior rostrum, mandibles present

**Previously Referred Specimens:** USNM V 15839 (Ballew, 1989); PEFO 31219 (Stocker, 2010); NMMNHS P-4239, 4256; YPM 3293 (Hunt *et al.*, 2006).

**Specimen(s) Used for Scoring:** UCMP 126999; PEFO 31219; PEFO 34853

**Key References:** Ballew (1989); Hunt *et al*. (2006)

**Most Recent Diagnosis:** Ballew (1989) diagnosed *Machaeroprosopus mccauleyi* based on the following autapomorphies: 1) Squamosal with distinct triangular outline without knob-like process; 2) Lateral portion of opisthotic thin and elongate; 3) Posttemporal fenestra large because of a medial expansion; 4) Basioccipital head relatively large; 5) Basioccipital neck relatively short.

**Modified Diagnosis:** 1) Squamosal with distinct triangular outline without knob-like process; 2) rostrum is completely crested in lateral view; 3) Ventral expansion of the alveolar rim at the border of the premaxillae and maxillae 4) Pre-infratemporal shelf does not extend under the posterior corner of the antorbital fenestra; 5) Anteroposterior corners of the antorbital fenestra rounded; 6) Anteromedial portion of the supratemporal fenestrae remain visible in dorsal view.

**Comments:** Characters 2, 3, 4 and 5 of Ballew (1989) do not appear to be different to those in other specimens of *Machaeroprosopus*. Character 1 does appear to be valid, but may be subject to intraspecific variability; PEFO 31219 (referred by Stocker, 2010) does possess a short, robust terminal knob on the posterior process of the squamosal, though in all other respects greatly resembles UCMP 126999 (the holotype).

Hunt *et al*. (2006) referred three specimens from Bull Canyon, New Mexico to *Machaeroprosopus mccauleyi* and cited three characters which link them to the holotype. Of the three skulls referred, we tentatively accept only one assignment (YPM 3293 [although the considerable robusticity of the specimen in comparison to the holotype complicates assignment]); we consider NMMNHS P-4256 to more likely represent ‘*Redondasaurus*’ *bermani* while NMMNHS P-4239 was not figured and was not examined here. Regardless, the suggested characters are relevant to *Machaeroprosopus mccauleyi* and differentiate this taxon from many other members of *Machaeroprosopus*, albeit not from ‘*Redondasaurus*’ *bermani*. Given the sparsity of robust phylogenetic characters given by Ballew (1989), the character suggestions of Hunt *et al.* (2006) are worth investigating. The first character is the same as character 1 of Ballew (1989), the others are: 2) In posterior view, the lateral margins of the skull flare at about 60°; 3) In lateral view, the rostrum is completely crested (inferred from the gradient of the holotype of *Machaeroprosopus mccauleyi* which lacks the distal rostrum). Character 2 of Hunt *et al*. (2006) is not diagnostic, being present in all robust species of *Machaeroprosopus* and may be prone to taphonomic distortion, and/or ontogenetic changes. Character 3 is useful as no other current species of *Machaeroprosopus* share this character. Neither character 1 of Ballew (1989), nor character 3 of Hunt *et al.* (2006) allow distinction of *Machaeroprosopus mccauleyi* from ‘*Redondasaurus*’ *bermani*; therefore, we suggest four further characters (our characters 3–6) to allow this differentiation.

***Machaeroprosopus buceros*** (Cope, 1881)

**Age:** late Norian–early Rhaetian (*c.* 213–207 Mya)

**Occurrences:** Petrified Forest Member, Chinle Formation, Orphan Mesa, New Mexico, USA; Upper Petrified Forest Member, Chinle Formation, Canjilon Quarry, New Mexico, USA; Upper Petrified Forest Member, Chinle Formation, Snyder Quarry, New Mexico, USA; Upper Cooper Canyon Formation, Dockum Group, Garza County, Texas, USA

**Holotype:** AMNH FR 2318, poorly preserved skull missing anterior end of rostrum

**Previously Referred Specimens:** UCMP 27228, 34246 & 34258; MNA V3478, CMNH 69727 (holotype of ‘*Redondasaurus*’ *bermani*) (Ballew, 1989); UCMP 27149 & 34250; UW 3807; MNA Pl. 25; NMMNHS P-18191, 31292, 33662, 33667, 33846, 33849, 33935, 35366, 35444, 35982, 36000, 36051, 36829, 37283, 37894 & 39700; FMNH UC 396 (holotype of ‘*Machaeroprosopus*’ *andersoni*); YPM 3293; TTU-P 09234 (Long & Murry, 1995); UCMP 27231, 27234, 34245 & 34249; GR 147 (Zeigler *et al.*, 2003a).

**Specimen(s) Used for Scoring:** AMNH FR 2318; TTU-P 11423; UCMP 34250; NMMNHS P-39700

**Key References:** Cope (1881); Ballew (1989); Long & Murry (1995); Lucas *et al.* (2002); Zeigler *et al*. (2003a, b)

**Most Recent Diagnosis:** Long & Murry (1995) diagnosed *Machaeroprosopus buceros* as a ‘heavy-skulled’ ‘pseudopalatine’ with the following combination of characters: 1) Squamosal with posterior process elongated, but deeper and shorter than that of *Pseudopalatus* [*Machaeroprosopus pristinus*], tapering into a blunt apex; 2) Descending process of squamosal large; 3) Rostrum partially crested; 4) Length shorter than posterior portion of skull; 5) Snout does not descend abruptly immediately anterior to external nares; 6) The latter are raised above the level of the skull roof as in *Pseudopalatus* [*Machaeroprosopus pristinus*]; 7) Crest sharp-edged with no sculpturing; 8) Dentition heterodont; 9) Alveoli closely spaced; 10) Enlarged anteriormost teeth and with dagger-like teeth at mid-length of premaxilla; 11) Crest deepest posteriorly.

**Modified Diagnosis:** In this study, we diagnose *Machaeroprosopus buceros* on the basis of the following unique character combination: 1) Posterior process of the squamosal is elongate and knob-like; 2) Tubular anterior portion of the rostrum has a triangular, rather than semi-circular, cross-section (amended from Ballew [1989] character 56); 3) Rostrum partially crested; 4) Snout does not descend abruptly immediately anterior to external nares.

**Comments:** Characters 1, 2, 4, 6, 8 and 9 of Long & Murry (1995) present problems; characters 1 and 2 describe morphologies that vary between specimens of *Machaeroprosopus buceros* (AMNH FR 2318, TTU-P 11423, UCMP 34250). The morphologies described by characters 4 and 6 disagree with the first-hand observations made in this study; with the rostrum measured from the most anterior point of the snout to the anterior border of the nares, no specimens observed here possessed a rostrum shorter in length than the narial + postnarial region of the skull - including even the holotype in which the most anterior section of the snout is lost. As in other species of *Machaeroprosopus* such as *Machaeroprosopus pristinus* and *Machaeroprosopus mccauleyi*, we found no evidence for the nares exceeding the height of the skull roof. Characters 8 and 9 describe features that are common to some extent in many, if not most, leptosuchomorph phytosaurs, and within *Machaeroprosopus* they are shared with *Machaeroprosopus mccauleyi*.

In their revision of North American phytosaurs, Long & Murry (1995) erected the new genus ‘*Arribasuchus*’, referring to it both *Machaeroprosopus buceros* and *Machaeroprosopus mccauleyi*. In a similar manner to their diagnosis of *Smilosuchus* the diagnostic character combination for the genus ‘*Arribasuchus*’ is the same as that for the type species, in this case *Machaeroprosopus buceros*. *Machaeroprosopus mccauleyi* was tentatively retained in ‘*Arribasuchus*’ by Long & Murry (1995), despite characters such as ‘rostrum partially crested’ being directly inconsistent with the species diagnosis of *Machaeroprosopus mccauleyi* given by Ballew (1989) (also used by Long & Murry to diagnose *Machaeroprosopus mccauleyi*).

Subsequent analyses have suggested that ‘*Arribasuchus*’ is paraphyletic (Hungerbühler, 2002; Hungerbühler *et al*., 2013; Parker & Irmis, 2006), and should be considered a junior synonym of *Machaeroprosopus* (Hungerbühler *et al*., 2013).

***Macheroprosopus pristinus*** (Mehl, 1928)

**Age:** late Norian–early Rhaetian (*c.* 213–207 Mya)

**Occurrences:** Chinle Formation, Apache County, Arizona, USA; Upper Petrified Forest Member, Chinle Formation, Canjilon Quarry, New Mexico, USA; Upper Petrified Forest Member, Chinle Formation, Snyder Quarry, New Mexico, USA

**Holotype:** MU 525, skull with many areas of plaster restoration

**Previously Referred Specimens:** UCMP 27018, 27235, 34245, 34249, 34251, 119436 & 131331; AMNH FR 7222; MNA V3495 (Ballew, 1989); NMMNHS P-31292; AMNH/GR 1027; UCM 55163; PEFO 4852; UCMP 27159 (referred to ‘*M*’. *zunii* by Camp [1930] and used for scoring ‘*M*’. *zunii* by Stocker [2010]; however, in the latter study this specimen is also referred to *Machaeroprosopus pristinus*, being mistakenly presented as the holotype of ‘*Machaeroprosopus tenuis*’. The correct specimen would be UCMP 27018), UCMP 27231, UCMP 27234; YPM 3294 (holotype of ‘*Redondasaurus*’ *gregorii*) (Long & Murry, 1995).

**Specimen(s) Used for Scoring:** MU 525; AMNH FR 7222; NMMNHS P-50040; PEFO 382; UCMP 137319; UCMP 27018 (‘*Machaeroprosopus tenuis*’ holotype)

**Key References:** Mehl (1928); Long & Murry (1995); Zeigler *et al.* (2002, 2003a, b)

**Most Recent Diagnosis:** Stocker (2010) used the following characters to diagnose *Machaeroprosopus pristinus*: 1) Supratemporal fenestrae nearly completely closed in dorsal view by medially expanded postorbital-squamosal bars, and the fenestrae are completely depressed below the level of the skull roof; 2) Squamosal process of the parietals immediately posterior to the main body of the parietals drop ventrally before continuing on to articulate with the parietal processes of the squamosals; 3) The posterior processes of the squamosals are expanded posteriorly as in *Leptosuchus*; however, there is no dorsoventral expansion of this posterior process, which is usually described as ‘knob-like’ in this taxon.

**Modified Diagnosis:** We use a combination of characters from Stocker (2010), one modified from Ballew (1989), and other novel characters: 1) Proportionally long rostrum (ratio of pre-narial to narial + post-narial length [measured to the posterior extremity of the parietals] greater than or equal to 2.2); 2) Subtriangular antorbital fenestra; anterior corner is pointed/acutely rounded and posterior border is taller and straight/gently rounded; 3) Rostrum descends immediately anterior to external nares and remains low and tubular for the majority of its length; 4) Tubular portion of rostrum is semi-circular in cross-section; 5) Weak heterodonty.

**Comments:** In her phylogenetic analysis, Stocker (2010) did not score *Machaeroprosopus pristinus* using the holotype, and instead used UCMP 27159 and NMMNHS P-31292. UCMP 27159 is a referred specimen of ‘*M*’. *zunii* (Camp, 1930) and was used as a referred specimen to phylogenetically score ‘*M*’. *zunii* in Stocker’s analysis; however, Stocker reported the same specimen number as the holotype of *Machaeroprosopus tenuis*, which was referred to, and used to score *Machaeroprosopus pristinus*. We assume this was a typographical error, and that Stocker actually scored and referred UCMP 27018 (actual holotype of ‘*Machaeroprosopus tenuis’*) to *Machaeroprosopus pristinus*, as UCMP 27159 is clearly different from *Machaeroprosopus pristinus* and UCMP 27018, based both on morphology and preservation. The choice to refer, and use NMMNHS P 31292 for scoring is puzzling; the skull displays a partial rostral crest which rises above the level of the nares and abruptly descends approximately at the midpoint of the external nares. No other referred specimen of *Machaeroprosopus pristinus* has a rostral crest, and the crest morphology is unknown in any other species of phytosaur. The specimen has previously been referred to *Machaeroprosopus buceros* (Zeigler *et al.*, 2002), to which *Machaeroprosopus pristinus* was also referred as a junior synonym and may explain the use of NMMNHS P-31292 to define *Machaeroprosopus pristinus* by Stocker. However, Stocker clearly stated that her analysis would not investigate the synonymy of these species, and in accordance used the proposed junior synonym ‘*Machaeroprosopus pristinus*’ in her analysis, demonstrating that no synonymy was assumed. Furthermore, Zeigler *et al*. (2002) attributed the unusual crest of NMMNHS P-31292 to post-mortem deformation; however, upon close study we believe the morphology to be genuine, which if true, casts uncertainty over the referral of this specimen to any currently known species.

The analysis of Hungerbühler *et al.* (2013) agreed with the hypothesis of these taxa being conspecific, with morphological differences due to sexual dimorphism; however no synapomorphies were given in support of this, and is therefore difficult to interpret. Hungerbühler *et al.* (2013) ultimately decided not to synonymise the taxa and urged against this action without “substantial supporting evidence”.

The diagnosis of Stocker (2010) does not allow differentiation of *Machaeroprosopus pristinus* and *Machaeroprosopus buceros*; in our revised diagnosis we therefore highlight that *Machaeroprosopus pristinus* possesses only weak heterodonty, whereas *Machaeroprosopus buceros* is strongly heterodont. This feature, however, is not included in our analysis character matrix.

***Machaeroprosopus lottorum*** Hungerbühler *et al*., 2013

**Age:** late Norian (*c.* 218–208 Mya)

**Occurrences:** Upper Cooper Canyon Formation, Dockum Group, Texas, USA

**Holotype:** TTU-P 10076, skull

**Previously Referred Specimens:** TTU-P 10077 (Hungerbühler *et al.*, 2013).

**Specimen(s) Used for Scoring:** TTU-P 10076; TTU-P 10077

**Key References:** Hungerbühler *et al*. (2013)

**Most Recent Diagnosis:** Hungerbühler *et al*. (2013) diagnosed *Machaeroprosopus lottorum* with the following characters: 1) Lateral rim of the naris broad, flat and rugose; 2) Supratemporal fenestra fully closed in dorsal aspect, forming a shallow semi-circular indentation into the skull roof, with a strongly bevelled rim that continues onto the parietal; 3) Free section of the postorbital/squamosal bar short; 4) Strongly developed horizontal medial laminae of palatines, that almost close the posterior section of the palatal vault in ventral view.

**Modified Diagnosis:** 1) Lateral rim of the naris broad, flat and rugose; 2) Supratemporal fenestra fully closed in dorsal aspect, forming a shallow semi-circular indentation into the skull roof, with a strongly bevelled rim that continues onto the parietal; 3) Strongly developed horizontal medial laminae of palatines, that almost close the posterior section of the palatal vault in ventral view.

**Comments:** We generally agree with the characters proposed by Hungerbühler *et al*. (2013), with the exception of their character 3. In TTU-P 10076 character 3 holds true i.e. the free section of postorbital/squamosal bar is proportionately shorter than in almost all other phytosaur specimens studied. However, in TTU-P 10077 the length of the free section of postorbital/squamosal bar is greater than in some specimens of *Machaeroprosopus pristinus* (UCMP 34249, 27231, 34228) including the holotype (MU 525), but shorter than other referred specimens (NMMNHS P-50040; PEFO 382; AMNH FR 7222); this character is also shorter in the majority of specimens of *Mystriosuchus planirostris*. This suggests the feature may be more variable than previously realized in *Machaeroprosopus lottorum* and is therefore removed from the diagnosis.

**‘*Redondasaurus*’ *gregorii*** Hunt & Lucas, 1993

**Age:** Rhaetian (*c.* 208.5–201.3 Mya)

**Occurrences:** Redonda Formation, Dockum Group, Shark Tooth Hill, Quay County, New Mexico, USA

**Holotype:** YPM 3294, poorly preserved and compressed skull missing left quadrate area, dorsal narial area and tip of rostrum

**Previously Referred Specimens:** OMNH 1250 (Hunt & Lucas, 1993).

**Specimen(s) Used for Scoring:** YPM 3294

**Key References:** Gregory (1972); Hunt & Lucas (1993); Hungerbühler *et al*. (2013)

**Most Recent Diagnosis:** Spielmann & Lucas (2012) built on the original diagnosis of Hunt & Lucas (1993) and diagnosed *Redondasaurus gregorii* using the following character combination: 1) Supratemporal fenestrae concealed in dorsal view; 2) Reduced antorbital fenestrae; 3) A prominent pre-infratemporal shelf at the anteroventral margin of the lateral temporal fenestra; 4) Septomaxillae that wrap around the outer margin of the external narial opening; 5) A thickened orbital margin; 6) An inflated posterior nasal behind the external narial opening; 7) Thickened dorsal osteoderms.

**Modified Diagnosis:** We retain most of the characters proposed by Spielmann & Lucas (2012) but reword them for more precise interpretation: 1) Supratemporal fenestrae concealed in dorsal view; 2) Antorbital fenestra with a distinct sharp corner at the anterior-most and posterior-most extremities; 3) Pre-infratemporal shelf projects anteriorly as a lobe reaching beneath the posterior corner of the antorbital fenestra, and dorsally joins with a ventrally descending flange of the postorbital; 4) Posterior border of the orbit equal to- or thicker than the dorsoventrally thinnest part of the posterior process of the jugal; 5) An inflated posterior nasal behind the external narial opening; 6) Postorbital/squamosal bars wide; 7) Thickened dorsal osteoderms.

**Comments:** We find issues with characters 2 and 4 of the diagnosis of Spielmann & Lucas (2012). The reduction of the antorbital fenestra in ‘*Redondasaurus*’ appears to be subjective based on the relative size of the antorbital fenestra when compared to the robusticity and size of the skull. In large specimens such as NMMNHS P-4256 and NMMNH P-31094 that have previously been referred to ‘*Redondasaurus*’, the antorbital fenestra appears small; however, in the holotype of ‘*Redondasaurus*’ *gregorii* (YPM 3294) the antorbital fenestra appears of similar proportions relative to the skull as in other phytosaurs such as *Mystriosuchus planirostris* or *Machaeroprosopus pristinus*. We suggest instead that the shape of the antorbital fenestra is unique in ‘*Redondasaurus*’ as both its anterior and posterior apices are sharp, rather than rounded; the antorbital fenestra only appears to be relatively small in specimens of ‘*Redondasaurus*’ *bermani* and is therefore used as a character for that species only. Although this is a generic feature of ‘*Redondasaurus*’ it is retained in this species diagnosis in case ‘*Redondasaurus*’ is synonymized with *Machaeroprosopus.* In such a scenario this character would be useful as part of a character combination to differentiate the species from almost all other members of the genus. We find no evidence for ‘septomaxillae’ that wrap around to the lateral side of the nares; Stocker (2010) found this feature to be present in both ‘*Redondasaurus*’ and *Pravusuchus hortus*; however, in the holotype of the latter this area is covered with iron oxide and may actually be the paranasal suture, which was identified in *Machaeroprosopus lottorum* by Hungerbühler *et al.* (2013). Given the phylogenetic proximity of *Machaeroprosopus lottorum* and ‘*Redondasaurus*’ it is likely that the feature described in ‘*Redondasaurus*’ may also be the paranasal; as the feature is currently ambiguous it is excluded from the diagnosis given here.

**‘*Redondasaurus*’ *bermani*** Hunt & Lucas, 1993

**Age:** Rhaetian (*c.* 208.5–201.3 Mya)

**Occurrences:** ‘siltstone member’, Chinle Formation, *Coelophysis* Quarry, New Mexico, USA

**Holotype:** CMNH 69727, skull

**Previously Referred Specimens:** Hungerbühler (2002) used a silhouette of NMMNHS P-4256 in their phylogeny to denote ‘*Redondasaurus*’ *bermani*, but referred to as ‘NMMNHS P-5246’. This appears to be a typographical mistake. NMMNHS P-4256 is included as a separate OTU to ‘*Redondasaurus*’ in this study so its affinities can be tested.

**Specimen(s) Used for Scoring:** NMMNHS P-4983

**Key References:** Hunt & Lucas (1993); Hunt *et al.* (2006); Hungerbühler (2002); Hungerbühler *et al.* (2013)

**Most Recent Diagnosis:** Hunt & Lucas (1993) diagnosed ‘*Redondasaurus*’ *bermani* as a ‘*Redondasaurus* species that differs from others in possessing a rostrum with a partial crest’.

**Modified Diagnosis:** A species of ‘*Redondasaurus*’ with the following characters: 1) Full rostral crest extending from nares to the terminal rosette of the premaxilla; 2) Antorbital fenestra reduced in size relative to other taxa of similar size and robuusticity; 3) Length of the symphyseal portion of the mandible approximately half that of the post-symphyseal region.

**Comments:** ‘*Redondasaurus*’ *bermani* was synonymized with *Machaeroprosopus buceros* by Long & Murry (1995) as mentioned above, and was also synonymized with ‘*Redondasaurus*’ *gregorii* by Spielmann & Lucas (2012) who concluded that it was the male sexual dimorph. Due to the lack of evidence for synonymy we tentatively retain ‘*Redondasaurus*’ *bermani* as a distinct species, but a thorough re-description of the species would be of great benefit.

***Mystriosuchus westphali*** (Hungerbühler & Hunt 2000)

**Age:** middle–late Norian (*c.* 216–209 Mya)

**Occurrences:** Löwenstein Formation (middle Stubensandstein), Middle Keuper Subgroup, Baden-Württemburg, Germany

**Holotype:** GPIT 261/001, skull with left side slightly distorted

**Previously Referred Specimens:** GPIT 261/17/7 (Hungerbühler & Hunt, 2000); GPIT 2145.000, 2146.000 & 2156.000 (provisionally included) (Hungerbühler, 2002).

**Specimen(s) Used for Scoring:** GPIT 261/001; AMNH FR 10644

**Key References:** Huene (1909; 1911); Hungerbühler (1998; 2002); Hungerbühler & Hunt (2000)

**Most Recent Diagnosis:** Hungerbühler (2002) listed eight autapomorphies for *Mystriosuchus westphali* as follows: 1) Discrete snout crest at midlength of the premaxillae; 2) Semicylindrical alveolar ridges; 3) Posterior process of the squamosal absent; 4) Squamosal contacts the prootic anteriorly; 5) Supraoccipital reaches the post-temporal fenestra and borders its dorsomedial half; 6) Lobate extension of the vertically descending squamosal process of the parietal; 7) Post-temporal fenestra is reduced to a narrow slit; 8) Presence of a discrete ossification (orbitosphenoid) anterior to the laterosphenoid.

**Modified Diagnosis:** 1) Discrete snout crest at midlength of the premaxillae; 2) Squamosal contacts the prootic anteriorly; 3) Supraoccipital reaches the post-temporal fenestra and borders its dorsomedial half; 4) Post-temporal fenestra is reduced to a narrow slit; 5) Presence of a discrete ossification (orbitosphenoid) anterior to the laterosphenoid; 6) A sharp corner of bone extends into the antorbital fenestra at approximately the midpoint of the posterior border, giving the posterior border a ‘stepped’ appearance.

**Comments:** Based on subsequent analyses and first-hand examination of specimens, we exclude characters 2, 3 and 6 of Hungerbühler (2002) from this diagnosis. Character 2 is present in almost all phytosaurs; characters 3 and 6 are both observed in the holotype of *Mystriosuchus westphali*. However, a specimen found in the collections of the AMNH (AMNH FR 10644), which is referable to *Mystriosuchus westphali* (as a species of *Mystriosuchus* that possesses a distinct sharp crest at the midlength of the premaxilla and lacks the abrupt concave rise of the rostrum into a narial crest), differs in displaying neither of these states. In AMNH FR 10644 the posterior process of the squamosal shares the same morphology as *Mystriosuchus planirostris* and the ‘lobate extensions’ on the squamosal process of the parietal are absent. Furthermore, these lobate extensions have been found in an indeterminate specimen of *Machaeroprosopus* (either *Machaeroprosopus pristinus*, *Machaeroprosopus buceros* or *Machaeroprosopus lottorum*) by Hungerbühler *et al*. (2013), but not in any others, suggesting this state is likely intraspecifically variable in multiple taxa. A further character, relating to the antorbital fenestra, is added which is found in both the holotype and AMNH FR 10644 but in no specimens of *Mystriosuchus planirostris*.

***Mystriosuchus planirostris*** (Meyer, 1863)

**Age:** middle–late Norian (*c.* 216–209 Mya)

**Occurrences:** Löwenstein Formation (middle Stubensandstein), Middle Keuper Subgroup, Baden-Württemburg, Germany; Zorzino Limestone, Lombardy, Italy

**Holotype:** MCZ 1018, fragment of right pre-orbital (lectotype); MCZ 1019A, 1019B, 1019C; MCZ 1022A, 1022B, rostral and skull fragments, partial caudal centrum (paralectotypes)

**Previously Referred Specimens:** SMNS 9134 (Fraas, 1896); SMNS 10260 (McGregor, 1906); SMNS 11126(1) (Huene, 1911); GPIT 249/002 (Huene, 1909); AMNH FR 10644 (Witmer, 1997); SMF uncat (Drevermann, 1918); MBSN 2 (Pinna, 1987); NHMW 1986 0024 0001 (Buffetaut, 1993); SMNS 13007, 13240, uncat 180, uncat 183, uncat 184 (possibly SMNS 9900); SMNS uncat 397, uncat 205; GPIT 2074.000, 2149.002, 2149.003, 2150.000; MB.I.008.05 (Hungerbühler, 1998).

**Specimen(s) Used for Scoring:** SMNS 10260; SMNS 9900; SMNS 9134; SMNS 13240; SMNS 91574

**Key References:** Meyer (1863); Fraas (1896); Hungerbühler (1998; 2002); Hungerbühler & Hunt (2000)

**Most Recent Diagnosis:** Hungerbühler (2002) listed six autapomorphic characters to distinguish *Mystriosuchus westphali* from *Mystriosuchus planirostris*: 1) The rostrum is extremely elongated; 2) A subvertical slope results in a concave profile of the prenarial area from side to side; 3) The external nasal opening is subdivided into a posterior section facing dorsally, and a strongly inclined anterior section that opens anteriorly; 4) The raised anterior border of the supratemporal fenestra extends along the medial rim of the squamosal; 5) The parieto-squamosal bar is depressed by more than 30 per cent of the skull height; 6) A larger quadrate foramen is present in a round recess formed by quadratojugal and quadrate.

**Comments:** Hungerbühler (2002) provided a detailed and useful discussion of characters previously used to diagnose *Mystriosuchus planirostris*, giving reasons why they should now be excluded.

**Specimen-level OTUs**

**NMMNHS P-4781**

**Age:** early Norian (*c.* 225–218 Mya)

**Occurrence:** Los Esteros Member, Santa Rosa Formation, Santa Fe County, New Mexico, USA

**Notes:** This specimen consists of the right orbital plus postorbital region of a skull, though lacking any of the interior or posterior elements such as the braincase, occipitals or palatines. Hunt *et al.* (1993) assigned this specimen to *Angistorhinus* sp. based on a combination of features: 1) Supratemporal fenestrae at the level of the skull roof; 2) Squamosals project posteriorly; 3) Squamosal process (parietal/squamosal bar) is rounded.

**TMM 31100-1332**

**Age:** late Carnian–early Norian (*c.* 232–225 Mya)

**Occurrence:** ‘Otis Chalk Quarry 3’, Colorado City Formation, Dockum Group, Howard County, Texas, USA

**Notes:** Stocker (2013) mentioned this specimen in reference to ‘*Angistorhinus*-like specimens from the Otis Chalk localities’. The specimen consists of a complete cranium, infilled with sediment, though lacking an associated mandible. Although the surface preservation is relatively good, there are many cracks through the skull, which cause slight displacements in areas such as the rostrum. The temporal region of the skull is slightly compressed dorsoventrally, causing the squamosal posterior processes and parietal/squamosal bars to curve posteroventrally.

**USNM V 21376**

**Age:** late Carnian–early Norian (*c.* 232–225 Mya)

**Occurrence:** Base of the Dockum Group, three miles North of Otis Chalk, Howard County, Texas, USA

**Notes:** This specimen was figured in lateral view by Stocker & Butler (2013) (Figure 5d), as an example of the genus *Angistorhinus*. The preorbital portion of the specimen is preserved, as is an area of skull roof including the prefrontals, frontals and the anterior parts of the postfrontals and parietals. The posterior processes of the squamosals are also preserved, as is the occipital condyle and ventral parts of the quadrates; however almost all of the postorbitals, jugals, quadratojugals and anterior and ventral parts of the squamosals are modelled with plaster. Due to the plaster reconstruction, the orientation of the supratemporal fenestrae is incorrect; the proximal remnants of the parietal/squamosal bars preserved on the squamosals have been aligned with the reconstructed postorbital/squamosal bars, whilst the parietal/squamosal bars are reconstructed entirely from plaster mimicking the depressed temporal morphology of *Mystriosuchus* or *Machaeroprosopus*. The specimen also preserves the symphysial region of the mandible, the anterior portions of the two rami including approximately the anterior third of the mandibular fenestra, and part of the left articular and retroarticular process. The nares appear to be elevated well above the level of the skull roof, although their posterior extremity appears to be damaged and the skull roof may be slightly crushed. The specimen may also be slightly mediolaterally compressed.

**PEFO 34852**

**Age:** early Norian (*c.* 225–220 Mya)

**Occurrence:** Blue Mesa Member, Chinle Formation, Petrified Forest National Park, Arizona, USA

**Notes:** This specimen consists of a complete cranium which has been crushed laterally at an oblique angle such that the external elements of the left half of the skull retain their original morphology, whereas the right half is strongly dorsoventrally compressed.

Griffin *et al*. (2017) referred this specimen to *Smilosuchus adamanensis* based on the following characters from the matrix of Kammerer *et al*. (2015): 1) An antorbital fossa is absent (3-3); 2) A rostral crest is present but not continuous (18-1); 3) The interorbital-nasal area is concave (21-1); 4) There is a moderate posterior process of the squamosal (24-1); 5) The posterior process of the squamosal is expanded in lateral view, but not rounded (25-1); 6) The squamosal fossa extends to the posterior edge of the squamosal (30-0); 7) The supratemporal fenestrae are partially depressed (32-1); 8) The supratemporal fenestrae are mostly visible in dorsal view (33-1).

However, upon first-hand comparison of these character scorings with the holotype of *S. adamanensis* and specimens of other non-mystriosuchin leptosuchomorph taxa, we find that all the above character scorings, aside from number 5, may equally refer to *Leptosuchus crosbiensis.* Furthermore, we find that the score for character 2 does not reflect the rostral morphology of either the holotype of *S. adamanensis* or our referred specimen UCMP 170166; in both specimens there is no evidence of any rostral crest, i.e. the rostrum forms an unbroken, straight slope from the posterior border of the nares to the premaxillae, whereupon the rostrum becomes tubular. However, in PEFO 34852, previously referred specimens of *L. crosbiensis* (USNM V 15481, TMM 31173-120, TTU-P 09230), the holotype of *L. crosbiensis* (subtly) and the holotype of *Leptosuchus studeri* (the sister taxon to *L. crosbiensis* in the analysis of Stocker (2010)), the narial openings extend horizontally from their posterior border, and directly anterior to the nares the rostrum either continues horizontally or slopes slightly ventrally, before dipping more strongly ventrally and levelling out to form a tubular rostrum. Therefore, from the characters presented it is unclear whether this specimen actually represents *S. adamanensis*; for this reason we include the specimen here as a separate OTU so its affinities can be tested phylogenetically.

**NMMNHS P-4256**

**Age:** late Norian (*c.* 218–208 Mya)

**Occurrence:** lower Bull Canyon Formation, Dockum Group, Barranca Badlands, Quay County, New Mexico, USA

**Notes:** This specimen consists of a large skull, missing the majority of its right postnarial region, the entire palate and the posterior section of the right mandibular ramus. Similarly to PEFO 34852, the skull has been compressed at an oblique dorsolateral angle leaving the left half relatively free from deformation, whilst the right half is strongly compressed and sheared dorsally.

According to Heckert *et al.* (2001) this specimen was originally referenced in the PhD thesis of Hunt (1994b) as a ‘robust morph’ of ‘*Redondasaurus*’ *gregorii*. Subsequently it was used in the phylogeny of Hungerbühler (2002) to exemplify ‘*Redondasaurus*’ *bermani*, rather than the (at the time) unnumbered Carnegie Museum specimen assigned as the holotype of ‘*Redondasaurus*’ *bermani* by Hunt & Lucas (1993) (CMNH 69727). Hunt *et al.* (2006) then referred this specimen to *Machaeroprosopus mccauleyi*, as a male sexual dimorph of the species due to the difference in skull size and rostral robusticity between this specimen and the holotype of *Machaeroprosopus mccauleyi.* Their species referral was based on three characters: 1) Posterior squamosal process is sub-triangular and lacks a knob-like termination; 2) In posterior view, the lateral margins of the skull flare at about 60 degrees; 3) In lateral view the rostrum is completely crested. All of these characters (regardless of their legitimacy or usefulness) can also be found in ‘*Redondasaurus*’ *bermani*; however, Hunt *et al.* also based their identification on an assumption that two species of ‘brachyrostral’ phytosaurs were unlikely to have occurred simultaneously geographically and temporally. As detailed earlier, the genus ‘*Redondasaurus*’ was redefined by Spielmann & Lucas (2012), and more diagnostic characters were added; again, disregarding the legitimacy of these characters, many of them are applicable to NMMNHS P-4256, suggesting the need for the placement of this specimen to be tested more thoroughly.

**USNM V 17098**

**Age:** early Norian (*c.* 221–219 Mya)

**Occurrence:** ?Bluewater Creek Member, Chinle Formation, Apache County, Arizona, USA

**Notes:** USNM V 17098 is a poorly preserved partial skull and mandible that are dorsoventrally compressed. The skull lacks most of the left lateral postnarial elements, though preserves much of the right half, the palate and braincase. The mandible is largely complete, though aspects are fragmentary and lacks the anterior-most portion of the terminal rosette.

This specimen was referred to *Leptosuchus* sp. by Long & Murry (1995) and again by Heckert & Lucas (2003); however, the label with the specimen identifies it as *Machaeroprosopus zunii*, though no justification has been provided for any of these three identifications. By scoring this specimen phylogenetically it may be possible to more definitively constrain its position.

**NMMNHS P-31094**

**Age:** Rhaetian (*c.* 208.5–201.3 Mya)

**Occurrence:** Redonda Formation, Dockum Group, Apache Canyon, Quay County, New Mexico, USA

**Notes:** This specimen consists of an extremely robust cranium, missing the majority of the premaxillae and the anterior extremities of the maxillae. The skull is slightly dorsoventrally crushed and slightly sheared. Heckert *et al.* (2001) provided a short description of the skull, referring the specimen to ‘*Redondasaurus*’ sp. on the basis of comparisons with other taxa, which we summarize as four characters: 1) Supratemporal fenestrae that are depressed and concealed in dorsal view; 2) Antorbital fenestra ‘tiny’ relative to narial length; 3) Postorbital/squamosal bars are anteroposteriorly short; 4) Postorbital/squamosal bars are broad.

**MB.R. 2747**

**Age:** Rhaetian (*c.* 208.5–201.3 Mya)

**Occurrence:** lower Exter Formation, near Salzgitter, Lower Saxony, Germany

**Notes:** MB.R. 2747 represents the largest phytosaur specimen found in Europe, and consists of a strongly deformed skull preserved in 11 articulating and non-articulating fragments, a partial mandible preserved in four articulating fragments, multiple vertebrae and centra, partial scapulae and coracoids, a humerus, and a set of articulated osteoderms. The skull retains the majority of the rostrum up to the anterior corner of the antorbital fenestrae, the posterior process of the right maxilla and the main bodies of the left and right jugals with the anterior corners of the lateral temporal fenestrae, a postnarial portion of the skull roof including a section of the posterior narial border and a dorsal part of the right orbital rim, a relatively complete, but crushed, braincase with dorsal portions of the parietals preserved and a fragment of the left postorbital/squamosal bar. The mandible consists of a short posterior section of the symphysis, from which the two rami bifurcate; the left ramus extends posteriorly such that part of the mandibular fenestra is preserved, whilst the right ramus does not extend as far as the beginning of the fenestra. The surface preservation of the material is generally good, but is extensively fractured making sutures difficult to discern.

This specimen was originally described by von Huene (1922) and was referred to the species ‘*Angistorhinopsis ruetimeyeri*’. This referral was based entirely on stratigraphic age and the size of the specimen, as the holotype of ‘*A. ruetimeyeri*’ consists of a partial phytosaur basioccipital, mandibular and postcranial fragments from a bonebed in Switzerland - none of which are diagnostic. The taxon ‘*A. ruetimeyeri*’ is therefore a nomen dubium; furthermore, MB.R. 2747 has never before been included in a phylogenetic analysis of phytosaurs. Its inclusion here will therefore provide a phylogenetic placement that may be useful in any future redescription of the specimen.

**NHMW 1986 0024 0001**

**Age:** middle–late Norian (*c.* 216–209 Mya)

**Occurrence:** Dachsteinkalk, Totes Gebirge, Styria, Austria

**Notes:** This specimen is an undescribed right half of a phytosaur skull from Austria, with a possibly associated partial mandible and ilium, that was referred to *Mystriosuchus planirostris* by Buffetaut (1993). Aside from the anterior tip of the snout and the quadratojugal, the half skull is relatively complete and well preserved with some sutures discernible; however, it may be somewhat mediolaterally compressed. The mandible is more poorly preserved; its dorsal surface is heavily weathered and the posterior half of the left ramus is missing, although the ventral surface is retained, allowing a more accurate estimate of skull length. Approximately the posterior quarter of the right ramus is missing. Similarly to MB.R. 2747, this specimen has never before been analysed phylogenetically, and its inclusion may assist future descriptive work.

**Appendix 2: Character list**

It is important to note here that when incorporating continuous and geometric morphometric character scorings for analysis, the format of the TNT data file requires these characters to be presented first in the file. This differs from how the characters are ordered in the character list below. Our character list presents characters in the order in which they occur for the base discrete matrix; where a character possesses a continuous or GM variant this is flagged next to that character, as indicated below. It should also be noted that characters in a TNT file begin at zero, whereas we shift our characters such that the list begins at one.

*** Character possesses a corresponding continuous variant**

**† Character possesses a corresponding/partially corresponding GM variant**

**1) Anterior end of premaxillae [from Stocker 2010, character 7]**

0: In anteroposterior plane of posterior rostrum

1: downturned

Although the distal terminus of the rostrum is downturned in all phytosaurs, in some such as *Parasuchus* and some specimens of *Machaeroprosopus pristinus*, there is dorsoventral constriction of the rostrum just posterior to the terminal rosette subsequent to which the rostrum deepens again such that the ventral edge is approximately level with the downturned anterior tip.

**2) Interpremaxillary fossa [Hungerbühler 2002, character 43; Stocker 2010, character 8]**

0: Absent

1: Present, broad and rounded

2: Present, narrow slit

Only species of *Mystriosuchus* display a narrow, slit-like fossa between the alveolar ridges; all other phytosaurs possess a broadly rounded fossa.

**3) Alveolar ridges [modified from Stocker 2010, character 9]**

0: Continuously visible in lateral view

1: Inconsistently visible, or entirely hidden in lateral view

Modified such that the state differences reflect the development of any kind of ventral overhang of the ventral rostral margin, rather than separating only those taxa in which such a ventral overhang is complete from those that display either an intermediate state or no overhang.

**4) Ventral alveolar bulge between premaxilla and maxilla [modified from Hungerbühler *et al.* 2013, character 2]**

0: Absent

1: Present

Wording modified for clarity. This ventral bulge of the tooth-row is consistently visible in *Smilosuchus gregorii* and most robust members of *Machaeroprosopus*. In some other taxa such as *Smilosuchus lithodendrorum*, *Pravusuchus hortus* and ‘*Redondasaurus*’ *gregorii* the bulge is not present in all specimens.

**5) Alveolar rim of maxilla [modified from Hungerbühler 2002, character 3; Stocker 2010, character 10]**

0: Horizontal or subconvex

1: Strongly ventrally convex

Wording altered slightly for clarity.

**6) Premaxillary crest [modified from Hungerbühler 2002, character 48]**

0: Absent

1: Present, rounded

2: Present, sharp

In the majority of phytosaurs an isolated premaxillary crest is absent, however *Mystriosuchus westphali* possesses a premaxillary crest with a sharp dorsal edge, giving the rostrum a more triangular coronal cross-section through the crest. *Leptosuchus studeri* and *crosbiensis* both also display an isolated premaxillary crest, however the dorsal edge is rounded, maintaining a curved dorsal profile in cross-section. This character is modified here to account for the different crest morphologies.

**7) Rostral crest [modified from Stocker 2010, characters 17, 18, 19]**

0: Absent

1: Narial crest a relatively abrupt rise to the nares interrupting a straight profile from rostrum to orbit

2: A straight steep slope from the nares to the premaxilla

3: Extends horizontally level from the nares for the majority of the crest with a terminal anterior slope

4: Extends horizontally level from nares for less than half the rostral length then descends and becomes tubular

This character was previously three separate characters, the first of which pertained to the morphology of the premaxilla, while the subsequent two scored the presence or absence of a ‘rostral crest’ and its morphology. Putting aside disputes over the cladistic usefulness of crest characters, these characters appear to overlap, risking artificial inflation of the influence of some traits. From the character state descriptions in the second and third characters, the ‘rostral crest’ appears to refer to the crest across both the premaxilla and maxilla. State zero of the first character (premaxilla dorsoventrally taller than mediolaterally wide) therefore directly overlaps with the second state of the next character (presence of a rostral crest). State one of the first character (tube-like morphology of the premaxilla) does not completely correlate with state zero of the next character (absence of rostral crest - rostrum tube-like for entire length) as morphologies exist (e.g., *Leptosuchus crosbiensis* and *studeri*) where the premaxilla is slender, but rises into a crest posterior to its contact with the maxilla; this would be described by a combination of state one in the first character, and state zero in the second. This morphology is, however, given a distinct state of its own in the third character: state zero (rostral crest partial or undulating from nares to terminal rosette); this state correlates exactly with a combination of states of the previous two characters. Furthermore, the third character is only applicable to taxa with rostral crests; un-crested taxa must therefore be scored as inapplicable which is treated as uncertainties during character optimization, resulting in their morphologies being ‘estimated’ for a trait they do not possess. Here we present a multi-state combination of the previously used characters, in which states are mutually exclusive and that is applicable to all taxa. An example of character state one is the abrupt rise to the nares in *Mystriosuchus planirostris*; state two is exemplified by *Smilosuchus gregorii*; state three is autapomorphic for *Nicrosaurus kapffi* and state four applies to taxa such as *Leptosuchus crosbiensis* and *studeri*.

*** [ORDERED] 8) Transverse width of the rostrum between the antorbital fenestrae in dorsal view [modified from Butler *et al.* 2014, character 46]**

0: Less than or equal to 1.20

1: 1.21 to 1.59

2: Greater than or equal to 1.60

States are here modified to represent the greater range of morphologies measured in this study. Measured as the ratio of the width of the rostrum between the antorbital fenestrae at their midpoint, and the interorbital distance at its shortest point. State zero corresponds to a narrow width, state one to moderate, and state two to a large width.

**9) Suture between maxilla, premaxilla and nasal [from Hungerbühler 2002, character 2]**

0: Slopes anteroventrally

1: Dorsally convex lobe

**10) Posterior portion of maxilla lateral outline in dorsal view [from Hungerbühler 2002, character 4]**

0: Straight/subconcave

1: Convex

*** [ORDERED] 11) Ratio of rostral to narial plus post narial length [modified from Hungerbühler 2002, character 1; Stocker 2010, character 14]**

0: Less than or equal to 1.50

1: 1.51 to 1.99

2: Greater than or equal to 2.00

In previous analyses this character used the pre-orbital and orbital + post-orbital lengths; however, orbital + post-orbital length was measured to the posterior process of the squamosal - the morphology of which is highly variable, and the subject of a number of other characters in their matrices. To avoid mixing the signal of this character with those of characters pertaining to the squamosal, we use the posterior extremity of the parietals as our posterior measuring point. The nares are used here rather than the orbits as phylogenetic signal is either unclear or lost when pre-orbital length is compared to the orbital + postorbital length to the posterior tip of the parietals. This suggests that much of the signal previously found in this character may be linked to variation in the squamosals, combined with rostral variation. The position of the nares does shift between phytosaurs belonging to, and excluded from Mystriosuchinae and thus presents a partial correlation with one other character pertaining to this change in position. However this is here judged to be a more favourable option than correlation with the squamosals, which are far more variable than the position of the nares, are the subject of more characters and have traditionally been used as one of the main diagnostic features for different groups of phytosaurs.

**12) Narial openings [from Hungerbühler 2002, character 50]**

0: Dorsally or anterodorsally

1: Anterior section opens forward, posterior upward

**13) Narial openings B [from Sereno 1991, character P; Stocker 2010, character 1]**

0: Directed laterally

1: Directed dorsally

**[ORDERED] 14) Position of nares [from Hungerbühler 2002, character 10; Stocker 2010, character 2]**

0: Terminal

1: Non-terminal, posterior rim of nares in front of anterior rim of antorbital fenestra

2: Non-terminal, posterior rim of nares behind anterior rim of antorbital fenestra

**15) Anterior extent of septomaxillae [from Stocker 2010, character 12]**

0: Anterior to anterior tip of nasal

1: Posterior to or at level with anterior tip of nasal

**16) Narial outlets [from Hungerbühler *et al.* 2013, character 10]**

0: Absent

1: Present

This character refers to grooves exiting the anterior extremity of the external nares, often resulting from the anterior convergence of the lateral narial borders. Narial outlets are almost entirely pervasive throughout non-leptosuchomorph phytosaurs, but only occur in a handful of more derived taxa; specifically in *Machaeroprosopus lottorum* and some specimens of *Nicrosaurus meyeri*, *Mystriosuchus planirostris* and *westphali* and *Machaeroprosopus pristinus*.

**17) Dorsal rim of nares [from Hungerbühler 2002, character 9; Stocker 2010, character 20]**

0: At or below level of skull roof

1: Above level of skull roof

**18) Narial wing [from Hungerbühler *et al.* 2013, character 11]**

0: Present

1: Absent, narial opening closed anteriorly

State zero refers to a raised lateral rim of the external nares, which descends prior to the anterior border of the nares, often abruptly, leaving a roughly 90 degree corner at the anterodorsal oint of the lateral narial rim, exemplified in *Mystriosuchus planirostris*.

**19) Interorbital nasal area lateral view [modified from Hungerbühler 2002, character 14; Stocker 2010, character 21]**

0: Flat from orbit to nares

1: Posterior border of nares and anterior border of orbits dip down into a concavity

This character and the subsequent one were previously a single character, describing the morphology of the interorbital-nasal area. However the original description of the character and its states are confusing: ‘Interorbitonasal area: flat (0); convex (1). The area between the nares and the orbits is primitively flat and broad. In derived phytosaurs, the area is narrower, transversely round, and saddle-shaped because of the elevation of the nares and the orbital rims.’ Hungerbühler (2002). The character initially appears to be describing only the transverse profile of the interorbital-narial area, however at the end the phrase ‘saddle-shaped’ is used in relation to the concavity seen in some phytosaurs in lateral view caused by the raised posterior border of the nares and anterior border of the orbits. This suggests the character should be aimed at describing the full three-dimensional morphology of the area, however this laterally visible morphology is not represented in the character states. The character is split here, in order to allow representation of both the laterally visible profile (character 19) and transverse morphology (character 20).

Additionally, whilst the states of character 20 are roughly similar to their original wording, their applicability to some taxa is different. Previously all derived taxa were stated to possess an interorbital-nasal area that ‘is narrower, transversely round’; however all members of Mystriosuchini excluding *Mystriosuchus* (and *Nicrosaurus* if included within Mystriosuchini) possess a much broader area between the nares and orbits than the more basal taxa, which is transversely flat, prior to its lateral descent. The cross section of this morphology is roughly rectangular in dorsal profile, rather than the inverted U-shape present in *Leptosuchus-*like phytosaurs, *Rutiodon* and *Angistorhinus*. In *Parasuchus*-grade phytosaurs, the large anteroposterior extent of the interorbital-nasal area results in a varied transverse morphology depending on the position at which it is sampled. We therefore tentatively exclude *Parasuchus*-grade phytosaurs from this character

**20) Interorbital nasal area cross section [modified from Hungerbühler 2002, character 14; Stocker 2010, character 21]**

0: Flat and broad

1: Dorsally curved in cross section

[See notes for character 19]

**21) Infranasal recess [from Hungerbühler 2002, character 11]**

0: Absent

1: Present

**[ORDERED] 22) Antorbital fossa [from Hungerbühler 2002, character 12; Stocker 2010: character 3; Butler *et al.* 2014, character 3]**

0: Present lacrimal jugal and maxillary fossae touching

1: Present but reduced lacrimal jugal and maxillary fossae in contact dorsally but not ventrally

2: Present but reduced lacrimal jugal and maxillary fossae not touching

3: Absent

**23) Discrete row of anteroposteriorly extending nodes on the lateral surface of the jugal [from Butler *et al.* 2014, character 44]**

0: Absent

1: Present

**24) Jugal and antorbital fenestra [from Stocker 2010, character 4]**

0: Excluded from antorbital fenestra

1: Contributing to antorbital fenestra

*** [ORDERED] 25) Length of antorbital fenestra [modified from Hungerbühler *et al*. 2013, character 13]**

0: Less than 1.9 times naris length

1: Greater than or equal to 1.9 times naris length

Modified to reflect the range of morphology sampled in this study. Measured as the ratio between the length of the antorbital fenestra and the length of the external nares. State zero scores a relatively shorter antorbital fenestra, while state one scores a relatively longer antorbital fenestra.

**26) Broad median depression on dorsal surface of frontals near border with nasals [from Kammerer *et al.* 2015, character 47]**

0: Absent

1: Present

**27) Posterolateral margins of nares [modified from Kammerer *et al.* 2015, character 48]**

0: Relatively low without ornamentation or derived features

1: Swollen and rugose creating a distinct narial rim

2: Distinctly raised in lateral view forming a sharp triangular peak well above the surrounding skull roof

State two is added here to reflect the morphology of *Parasuchus bransoni*, which deviates from the previous two states with the dorsally pronounced morphology of its posterior narial rim.

**28) Pre-orbital depression [from Hungerbühler 2002, character 15]**

0: Absent

1: Present

**29) Depression and flange in postorbital bar [from Hungerbühler *et al.* 2013, character 14]**

0: Absent

1: Small elongate depression posterior rim of postorbital may create a small flange behind orbit

2: Strong elongate depression posterior rim of postorbital bar forms a distinct flange merging with po/sq bar

**30) Jugal and orbit [from Stocker 2010, character 5]**

0: Excluded from orbit

1: Contributing to orbit

In *Nicrosaurus kapffi*, both states of this character are present, with both states represented in individual specimens in some cases **(Hungerbühler, 1998)**.

**31) Medial margins of orbits [from Stocker 2010, character 6]**

0: Flat with skull roof

1: Raised into orbital ridges

**32) Deep sculpture of the skull roof [from Hungerbühler 2002, character 17]**

0: Absent

1: Present

**33) Sutural articulation of squamosal and postorbital in dorsal view [from stocker 2010, character 22]**

0: Slot like, posterior process of po fits into slot in sq

1: Diagonal, sq forms anteromedial portion of po/sq bar and po forms posterolateral portion

2: Approximately transverse

**[ORDERED] 34) Most anterior extent of infra-temporal fenestra [from Butler *et al.* 2014, character 45]**

0: Beneath the posterior corner of the orbit

1: Extended anteriorly, reaches below the middle or anterior half of the orbit

2: Anteroventral corner distinctly in front of anterior rim of orbit

**35) Pre-infratemporal shelf [from Hungerbühler 2002, character 18]**

0: Absent

1: Present

The ‘pre-infratemporal shelf’ is an anteriorly convex, crescent-shaped ridge slightly anterior of the anterior border of the antero-ventral corner of the infratemporal fenestra. This morphology is present in all members of *Mystriosuchus*, *Machaeroprosopus* and ‘*Redondasaurus*’ to some degree, and also in some specimens of *Nicrosaurus*.

**36) Lateral ridge from post-orbital/squamosal bar [modified from Stocker 2010, character 23; Butler *et al*. 2014, character 23]**

0: Absent

1: Continues posteriorly onto squamosal as a horizontal ridge forming a shelf overhanging the infratemporal fenestra

2: Bifurcates into two small ridges on lateral surface of squamosal

The morphology of any ridge on the lateral surface of the postorbital/squamosal bar has previously been scored with considerable subjectivity. The original character on which this is based possesses a number of states which may be equally applicable to multiple taxa depending on interpretation. Here, the character is simplified to reflect the morphologies that were recognized in this study, including the absence of a ridge.

**37) Lateral ridge of postorbital squamosal bar continues as ridge onto posterior process of squamosal [from Hungerbühler *et al.* 2013, character 19]**

0: Absent

1: Present

*** [ORDERED] 38) Length of posterior process of squamosal in relation to postorbital length [modified from Hungerbühler 2002, character 31; Stocker 2010, character 24]**

0: Absent or extremely short, posterior edge of squamosal does not extend or barely extends posteriorly beyond the distal end of the paroccipital process of the opisthotic

1: Less than 3.60

2: 3.60 to 4.99

3: Greater than, or equal to 5.00

Modified to reflect the measurements made for the greater range of taxa included in this study. The character is measured as the ratio of the distance from the posterior border of the orbit to the posteriormost point of the squamosal, and the distance from the posterior border of the paroccipital process to the tip of the squamosal. State one scores a long posterior process, state two a moderate process, and state three a short process.

**† 39) Posterior process of squamosal [modified from Hungerbühler 2002, character 32; Stocker 2010, character 25]**

0: Greatly dorsoventrally expanded

1: Moderately dorsoventrally expanded

2: Terminal knob

Ballew (1989), character 50 references the presence of a ‘knob-like’ posterior process of the squamosal in *Machaeroprosopus pristinus* and *buceros*; this then became the ‘terminal knob’ of Hungerbühler (2002) and was subsequently used to describe this morphology. This character is modified to use this terminology, which is assumedly referenced by the ‘dorsally compressed’ state, used in previous versions of this character. Using this terminology makes the character less ambiguous.

**† 40) Terminal knob [modified from Hungerbühler 2002, character 32; Hungerbühler *et al.* 2013, character 24]**

0: Terminal knob raised distally above po/sq bar

1: Terminal knob in plane of po/sq bar

In some specimens the distal region of the terminal knob-like process of the squamosal is inflexed dorsally. The previous version of this character mentioned the posterior raising of the posterior process of the squamosal in reference to a greatly dorsoventrally expanded posterior process. This morphology was not recognized in any specimens in this study, whereas it was noted to be relatively common among individuals possessing a terminal knob.

**41) Dorsal edge of squamosal [from Hungerbühler 2002, character 33; Stocker 2010, character 26]**

0: Straight and narrow, no medial expansion

1: Expanded medially

This character essentially scores the presence or absence of any size of medial flange of the postorbital/squamosal bar.

**42) Dorsal edge of squamosal B [from Stocker 2010, character 27]**

0: Mediolaterally flat

1: Ventral depression between medial and lateral edges of the dorsal edge of the squamosal

*** [ORDERED] 43) Length of free postorbital/squamosal bar [modified from Hungerbühler *et al.* 2013, character 17]**

0: Less than 2.90

1: 2.90 to 3.39

2: Greater than or equal to 3.40

Modified to reflect the measurements made for the greater range of morphologies in this study. The character is measured as the ratio between the distance from the most anterior point of the supratemporal fenestra and the posteriormost point of the squamosal, to the shortest distance between the posterior border of the orbit and the most anterior point of the supratemporal fenestra. State zero corresponds to ‘short’, one corresponds to ‘moderate’ and two to ‘long’.

**44) Medial extent of squamosal [modified from Hungerbühler 2002, character 30]**

0: To mid length of parieto squamosal bar

1: Enters base of supraoccipital shelf wedged between parietal and supraoccipital

The character state ‘Enters rim of supraoccipital shelf dorsal to parietal’ has been removed as it was not recognized in any of the specimens examined in this study.

**45) Cross section of posterior half of postorbito squamosal bar [from Hungerbühler 2002, character 22]**

0: Low, dorsoventrally compressed

1: High, triangular

**† 46) Ventral margin of squamosal [from Stocker 2010, character 28]**

0: Gently sloping anteroventrally from posterior edge of posterior process to opisthotic process

1: Distinct horizontal ventral edge between posterior edge of posterior process and opisthotic process

**47) Subsidiary opisthotic process of squamosal [from Hungerbühler 2002, character 35; Stocker 2010, character 29]**

0: Absent

1: Present

**48) Extent of squamosal fossa [from Stocker 2010, character 30]**

0: Extends to posterior edge of sq

1: Does not reach posterior edge of sq

**[ORDERED] 49) Orientation of supratemporal fenestra [from Stocker 2010, character 32]**

0: Dorsally expressed parietal squamosal bar at level with skull roof

1: Partially depressed parietal process of squamosal below level of skull roof

2: Fully depressed posterior process of parietal and entire parietal squamosal bar below level of skull roof

**† 50) Mediolateral expansion of posterior process of squamosal [modified from Hungerbühler *et al*. 2013, character 25]**

0: Tip of squamosal tapers strongly posteriorly

1: Tip of squamosal tapers with a smooth lateral deflection distally

**51) Face of medial rim of squamosal along supratemporal fenestra and posterior process [modified from Hungerbühler *et al*. 2013, character 23]**

0: Entire rim rounded or sharp

1: Rim entirely or in part squared

**52) Extent of squaring of the squamosal rim [modified from Hungerbühler *et al*. 2013, character 23]**

0: Squared in posterior section

1: Entire rim squared

**53) Ridge around anterior and or medial edge of supratemporal fenestra [modified from Hungerbühler 2002, character 20]**

0: Absent

1: Present, medial only

2: Present, anterior and medial

The terminology used in the original character is quite vague ‘Anterior border of supratemporal fenestra… raised above skull roof’. This character is a reinterpretation of the original, with more specific terms. In state one, the ridge would only be present on the parietal ledge, whereas in state two the ridge may extend to the anterior border of the supratemporal fenestra.

*** † [ORDERED] 54) Width of squamosal [modified from Hungerbühler *et al.* 2013, character 18]**

0: Less than or equal to 3.80

1: Greater than 3.80

Modified to reflect the greater range of morphologies measured in this study. Scored as the ratio between the length of the squamosal from the anteriormost point of the supratemporal fenestra to the posterior-most extent of the posterior process, and the width of the postorbital/squamosal bar at its approximate mid-point, or the point most representative of its general width. State zero corresponds to a wide postorbital/squamosal bar, state one denotes a relatively less wide bar.

**† 55) Outline of medial rim of squamosal along supratemporal fenestra and posterior process [modified from Hungerbühler 2002, character 29]**

0: Sinuous

1: Angular

2: Straight

3: Curved

Character state three has here been added to represent the morphologies found in *Nicrosaurus* and *Coburgosuchus*, which we feel were not adequately described by the previous character states.

**56) Path of parietal/squamosal bars [from Stocker 2010, character 34]**

0: Trending straight posteriorly to attachment on squamosals

1: Curved medially convex before attaching on squamosals

**[ORDERED] 57) Visibility of supratemporal fenestrae in dorsal view [modified from Hungerbühler 2002, character 19; Stocker 2010, character 33]**

0: Visible, STF completely open dorsally

1: Mostly visible, posterolateral portions of STF covered in dorsal view

2: Mostly covered dorsally, at most only anteromedial corners or medial slit of STF visible in dorsal view

3: Lamella merges with parietal, STF obliterated in dorsal view

This character combines those of Hungerbühler and Stocker. The wording used by Stocker is more inherently understandable, as Hungerbühler describes the visibility of the fenestra via the expansion of the squamosal, which is technically correct, but less intuitive. The final state of Hungerbühler’s character is, however, missing from Stocker’s and is useful in describing the morphology found in some extremely robust members of *Machaeroprosopus* and most specimens of ‘*Redondasaurus*’.

**58) Parietal/squamosal bars [from Stocker 2010, character 35]**

0: Slender, narrower than the width of po/sq bars

1: Wide, approximately the same mediolateral width as po/sq bars

**[ORDERED] 59) Dorsal edge of parietal/squamosal bar [from Hungerbühler 2002, character 26]**

0: Horizontal

1: Gently sloping

2: Steeply sloping

3: Either entirely, or in parts vertical

*** [ORDERED] 60) Parietal ledge, ratio of width to length [modified from Hungerbühler *et al.* 2013, character 30]**

0: Less than 1.30

1: 1.30 to 2.10

2: Greater than 2.10

Modified to reflect the greater range of morphologies sampled in this study. Width is measured either at the mid-point of the ledge, or the point that is most representative of its general width. Length is measured from the posterior-most extent of the ledge to the anterior-most point of the supratemporal fenestra (in phytosaurs where the parietal/squamosal bars are at the level of the skull roof, the posterior-most point of the ledge is measured at the midline of the parietals). State zero corresponds to a parietal ledge that is generally more anteroposteriorly prominent, whereas state two is very wide and hardly projects posteriorly.

**61) Medial half of parieto squamosal bar lateral wall of supraoccipital shelf [from Hungerbühler 2002, character 37]**

0: High and thin

1: Low, continuously thin

2: Low, basally thickened

**62) Lobate extension on the vertical rim of the squamosal processes of the parietal [from Hungerbühler 2002, character 53]**

0: Absent

1: Present

**63) Depth and shape of supraoccipital shelf [from Hungerbühler 2002, character 36]**

0: Shallow, longitudinal axis of shelf vertical

1: Deep, axis of shelf straight and horizontal

2: Deep axis of shelf with steep slope anteriorly and terminal horizontal deflection of shelf

**64) Top of parieto supraoccipital complex formed by squamosal processes of parietals [from Ballew 1989, character 19; Hungerbühler 2002, character 24]**

0: Angular, inverted V shape

1: Rounded, inverted U shape

2: Rectangular

**65) Parietal prongs [from Hungerbühler 2002, character 25]**

0: Absent

1: Present

**66) Posttemporal fenestra [from Hungerbühler 2002, character 41]**

0: Moderately wide and tall

1: Moderately wide and compressed

2: Extremely reduced in width and height to a slit

**67) Lateral border of posttemporal fenestra [Hungerbühler 2002, character 38; Stocker 2010, character 37]**

0: Formed by contact of the parietal process of the squamosal and the paroccipital process of the opisthotic

1: Formed laterally only by the paroccipital process

2: Formed laterally and slightly ventrally by process of squamosal that extends onto paroccipital process

**68) Shape of quadratojugal [modified from Sereno 1991, character Q; Stocker 2010, character 31]**

0: L shaped, anterior suture trends anteroventrally

1: Subtriangular

2: L shaped, anterior suture trends anterodorsally

**69) Anterior border of parabasisphenoid contribution to basitubera [from Stocker 2010, character 39]**

0: Basitubera separated widely

1: Basitubera separated narrowly with a ridge along their anterior border

2: Basitubera connected tubera form a sharp ridge along their anterior border

**70) Morphology of basioccipital between tubera [from Stocker 2010, character 40]**

0: Concave depression

1: Anteroposteriorly oriented ridge on the midline

**71) Lateral extent of basitubera compared to basipterygoid processes in ventral view [from Stocker 2010, character 41]**

0: Lateral extent of basitubera even with lateral extent of basipterygoid processes

1: Lateral extent of basitubera more laterally expanded compared to basipterygoid processes

**72) Length of interpterygoid vacuity [from Chatterjee 1978, phenetic feature 7; Hungerbühler 2002, character 47]**

0: Long, more than 50 per cent of length of palatal vault

1: Tiny oval indentation at posterior rim of conjoined pterygoids

**73) Suborbital foramen [from Hungerbühler 2002, character 46; Stocker 2010, character 43]**

0: Elongated, wide

1: Elongated, slit-like

2: Reduced to a single oval fenestra or subdivided into two or more small openings

**74) Anterior extent of the palatine [from Hungerbühler 2002, character 44]**

0: Tip located behind the posterior rim of choana

1: Tip extends forward beyond the posterior rim of choana

2: Tip extends forward beyond the anterior rim of choana

**75) Palatal ridge [from Hungerbühler *et al.* 2013, character 40]**

0: Low, rounded longitudinal elevation

1: Prominent, sharp ventrally to ventromedially directed crest

**76) Medial edge of palatine below posterior part of palatal vault [from Hungerbühler *et al.* 2013, character 41]**

0: Sloping or vertical

1: Horizontal flange may restrict the opening of the palatal vault significantly

**77) Dorsal surface of surangular [from Mateus *et al*. 2014]**

0: Gently convex

1: Gently concave rising to apex just posterior to dentary contact

**78) Shape of retroarticular process in lateral view [from Mateus *et al*. 2014]**

0: Distally sharply pointed or curved into a posterodorsally oriented hook

1: Distally rounded or blunt

**79) Snout dorsal surface cross sectional shape**

0: Rounded, dorsal surface of snout is curved from side to side

1: Triangular, sides of the snout are straight and slope up to the midline

**80) Anterior separation of the septomaxillae**

0: Septomaxillae separate posterior to level with the anterior narial border

1: Septomaxillae separate distinctly anterior of the anterior narial border

**† 81) Shape of antorbital fenestra**

0: Ellipsoid

1: Approximately a geometric ‘stadium’ shape

2: Approximately triangular

3: Approximately triangular - point posteriormost

**82) Lateral surface of maxilla and jugal ventral/posteroventral to AOF**

0: Flat/laterally convex

1: Concavity running along the length of the element

**83) Lateral surface of main body of jugal**

0: Generally flat, element forms one dorsolaterally facing plane between its ventral and dorsal extremities

1: Anteroposteriorly directed ridge running from below AOF towards ventral border of subTF splits jugal into a dorsolaterally facing facet and a laterally facing facet

2: Anteroposterior ridge running along the centre of the jugal posterior process

3: Anteroposterior ridge running toward ventral border of jugal posterior process

**84) Anterior extension of the pre-infratemporal shelf**

0: Anteriormost border of shelf is posterior to the posteriormost border of the antorbital fenestra

1: Anteriormost border of shelf terminates anterior of the posteriormost corner of the antorbital fenestra

**85) Dorsal extension of pre-infratemporal shelf**

0: Merges dorsally into lateral surface of jugal

1: Continues dorsally contributing to the posterior edge of the postorbital descending process

**86) Jugal foramen in anteroventral corner of the infra-temporal fenestra**

0: Visible only in medial view, not visible in lateral view

1: Visible in lateral view

*** [ORDERED] 87) Relative robusticity of the jugal**

0: Less than 7.30 (Robust)

1: 7.30-8.40 (Moderate)

2: Greater than 8.40 (Slender)

This ratio is calculated as the distance from the posterolateral-most corner of the quadrate to the anteroventral corner of the infra-temporal fenestra, divided by the thickness of the posterior process of the jugal at its thinnest point below the infra-temporal fenestra (measured perpendicular to the long axis of the element, i.e. approximately dorsoventrally).

**88) Proximal section of postorbital descending process where posterior border of orbit meets skull roof**

0: Flares anteroposteriorly creating a wide triangular connection

1: Posterior border of orbit remains thin until it reaches skull roof

*** † [ORDERED] 89) Infra-temporal fenestra diagonal aspect ratio**

0: Less than or equal to 2.30

1: Greater than 2.30

Measured as the distance from the anteroventral corner, to the posterodorsal corner of the infra-temporal fenestra, divided by the distance from the anterodorsal corner, to the posteroventral corner of the infra-temporal fenestra.

**90) Additional ridge on lateral surface of posterior process of squamosal below ridge or rugosity from po/sq bar**

0: Absent

1: Present

**† 91) Posterior border of quadrate in lateral view**

0: Straight anterodorsal line for majority of element

1: Ventral section of border is anteroposteriorly concave

**92) Internarial septum**

0: Restricted to between, or extends slightly anterior of the external nares

1: Extends anterior of the nares by approximately the narial length

**93) Triangular projection ventro-medial to the glenoid of the articular**

0: Anterior border is straight, trending posteroventrally

1: Ventral half of anterior border possesses an anteriorly projecting process

*** [ORDERED] 94) Relative length of mandibular symphysis**

0: Less than 1.00 (Short)

1: Greater than or equal to 1.00 (Long)

Measured as the anteroposterior length of the mandibular symphysis, divided by the anteroposterior length of the post-symphyseal region.

**Appendix 3: Nodal synapomorphies**

This list presents all synapomorphic character changes at every node of each of the four data treatments: discrete, discrete + continuous, discrete + GM, and discrete + continuous + GM.

**Discrete tree**

1) No synapomorphies

2) 13: 0→1

22: 0→1

3) 36: 0→1

69: 0→1

4) 23: 0→1

26: 0→1

50: 0→1

5) 82: 0→1

91: 0→1

6) 1: 0→1

22: 1→2

73: 0→1

83: 1→0

7) 9: 0→1

14: 1→2

19: 0→1

29: 0→1

36: 1→0

38: 0→2

69: 1→2

72: 0→1

74: 1→2

80: 0→1

81: 0→2

8) 22: 2→1

60: 1→0

92: 0→1

9) 56: 0→1

58: 0→1

10) 42: 0→1

51: 0→1

11) 12: 0→1

17: 0→1

66: 0→1

70: 0→1

12) 69: 2→1

13) 8: 0→2

16: 1→0

25: 0→1

41: 0→1

44: 0→1

49: 0→1

57: 0→1

59: 0→1

88: 1→0

14) 81: 2→1

15) 90: 0→1

16) 19: 1→0

20: 0→1

39: 0→1

17) 7: 0→4

18) 48: 0→1

19) 3: 0→1

7: 4→2

33: 0→2

20) 21: 0→1

29: 1→0

21) 49: 1→2

53: 2→0

54: 1→0

57: 1→2

67: 0→2

22) 38: 2→3

23) 49: 1→2

24) 35: 0→1

43: 2→0

54: 1→0

75: 0→1

87: 0→1, 2

25) 59: 1, 2→3

84: 0→1

85: 1→0

26) 2: 1→2

20: 0→1

27) 88: 0→1

28) 65: 0→1

29) 3: 0→1

4: 0→1

7: 1→4

46: 1→0

47: 0→1

30) 25: 1→0

48: 0→1

31) 30: 1→0

36: 0→1

51: 0→1

60: 1→2

32) 3: 1→0

4: 1→0

48: 0→1

90: 0→1

33) 7: 4→1

8: 2→1

89: 0→1

34) 7: 4→2

22: 2→3

35) 11: 1→0

36) 48: 1→0

53: 1→0

37) 19: 1→0

57: 2→3

59: 2→3

63: 1→0

38) 89: 0→1

**Discrete + continuous tree**

1) No synapomorphies

2) 13: 0→1

22: 0→1

3) 36: 0→1

69: 0→1

4) 23: 0→1

26: 0→1

50: 0→1

54: 0.525-0.545 → 0.353-0.420

5) 25: 0.452-0.470 → 0.409-0.446

82: 0→1

91: 0→1

6) 1: 0→1

11: 0.459-0.484 → 0.490-0.686

22: 1→2

25: 0.452-0.470 → 0.514

73: 0→1

83: 1→0

89: 0.244-0.272 → 0.457-0.520

7) 9: 0→1

14: 1→2

29: 0→1

36: 1→0

60: 0.060-0.090 → 0.036-0.038

69: 1→2

72: 0→1

74: 1→2

80: 0→1

8) 19: 0→1

22: 2→1

25: 0.514 → 0.503

92: 0→1

9) 56: 0→1

58: 0→1

87: 0.106-0.110 → 0.103

10) 42: 0→1

51: 0→1

11) 8: 0.240-0.253 → 0.274

12: 0→1

17: 0→1

66: 0→1

70: 0→1

12) 69: 2→1

13) 5: 0→1

16: 1→0

25: 0.514 → 0.684-0.718

41:0→1

88: 1→0

90: 0→1

14) 57: 0→1

87: 0.106-0.110 → 0.117-0.132

15) 10: 0→1

79: 0→1

16) 11: 0.678-0.686 → 0.729

54: 0.525-0.545 → 0.395-0.406

17) 48: 0→1

89: 0.457 → 0.462

18) 21: 0→1

29: 1→0

19) 49: 1→2

54: 0.395-0.406 → 0.270

57: 1→2

67: 0→2

20) 54: 0.270 → 0.159

89: 0.457 → 0.615-0.833

21) 19: 0→1

20: 1→0

22) 33: 0→1

38: 0.281 → 0.457

54: 0.525-0.545 → 0.494

67: 0→2

90: 1→0

23) 49: 1→2

54: 0.494 → 0.383-0.399

24) 43: 0.106-0.118 → 0.070

53: 2→1

54: 0.383-0.399 → 0.213

61: 0→2

65: 0→1

25) 59: 1→2

60: 0.098-0.102 → 0.125

26) 60: 0.125 → 0.236-0.267

27) 48: 0→1

28) 35: 0→1

38: 0.457-0.751 → 0.325-0.442

89: 0.507 → 0.550-0.582

29) 22: 3→2

75: 0→1

30) 39: 1→0

53: 1→2

31) 3: 1→0

4: 1→0

32) 39: 1→2

90: 0→1

33) 43: 0.070 → 0.034-0.043

48: 1→0

59: 2→3

86: 0→1

34) 25: 0.439-0.514 → 0.113

34: 1→2

53: 1→0

54: 0.180-0.197 → 0.039-0.095

89: 0.550-0.620 → 0.710

35) 84: 0→1

87: 0.338-0.401 → 0.574-0.577

36) 7: 1→2

53: 1→2

57: 2→3

37) 57: 2→1

61: 2→1

85: 1→0

38) 2: 1→2

20: 0→1

39) 19: 0→1

88: 0→1

**Discrete + GM tree (excluding landmark ‘state changes’)**

1) [39, 40, 46]: LANDMARK

[50, 54, 55]: LANDMARK

81: LANDMARK

89: LANDMARK

91: LANDMARK

2) 13: 0→1

22: 0→1

[39, 40, 46]: LANDMARK

[50, 54, 55]: LANDMARK

81: LANDMARK

89: LANDMARK

91: LANDMARK

3) 36: 0→1

[39, 40, 46]: LANDMARK

[50, 54, 55]: LANDMARK

69: 0→1

81: LANDMARK

89: LANDMARK

91: LANDMARK

4) 23: 0→1

26: 0→1

[39, 40, 46]: LANDMARK

[50, 54, 55]: LANDMARK

81: LANDMARK

89: LANDMARK

91: LANDMARK

5) [39, 40, 46]: LANDMARK

[50, 54, 55]: LANDMARK

81: LANDMARK

82: 0→1

89: LANDMARK

91: LANDMARK

6) 1: 0→1

22: 1→2

[39, 40, 46]: LANDMARK

[50, 54, 55]: LANDMARK

81: LANDMARK

83: 1→0

89: LANDMARK

91: LANDMARK

7) 11: 0→1,2

34: 0→1

42: 1→0

[39, 40, 46]: LANDMARK

[50, 54, 55]: LANDMARK

81: LANDMARK

89: LANDMARK

91: LANDMARK

8) [39, 40, 46]: LANDMARK

[50, 54, 55]: LANDMARK

81: LANDMARK

89: LANDMARK

91: LANDMARK

9) 9: 0→1

14: 1→2

[39, 40, 46]: LANDMARK

[50, 54, 55]: LANDMARK

80: 0→1

81: LANDMARK

89: LANDMARK

91: LANDMARK

10) 19: 0→1

22: 2→1

[39, 40, 46]: LANDMARK

81: LANDMARK

89: LANDMARK

91: LANDMARK

92: 0→1

11) [39, 40, 46]: LANDMARK

56: 0→1

58: 0→1

81: LANDMARK

89: LANDMARK

91: LANDMARK

12) 42: 0→1

[39, 40, 46]: LANDMARK

51: 0→1

81: LANDMARK

89: LANDMARK

91: LANDMARK

13) 12: 0→1

17: 0→1

66: 0→1

70: 0→1

81: LANDMARK

14) 69: 2→1

81: LANDMARK

15) 5: 0→1

16: 1→0

25: 0→1

[39, 40, 46]: LANDMARK

49: 0→1

[50, 54, 55]: LANDMARK

57: 0→1

59: 0→1

81: LANDMARK

89: LANDMARK

91: LANDMARK

16) 41: 0→1

[39, 40, 46]: LANDMARK

[50, 54, 55]: LANDMARK

81: LANDMARK

89: LANDMARK

91: LANDMARK

17) [39, 40, 46]: LANDMARK

48: 0→1

[50, 54, 55]: LANDMARK

81: LANDMARK

89: LANDMARK

90: 0→1

91: LANDMARK

18) [39, 40, 46]: LANDMARK

[50, 54, 55]: LANDMARK

81: LANDMARK

89: LANDMARK

91: LANDMARK

19) 3: 0→1

33: 0→2

[39, 40, 46]: LANDMARK

[50, 54, 55]: LANDMARK

81: LANDMARK

89: LANDMARK

91: LANDMARK

20) 29: 1→0

33: 0→1

[39, 40, 46]: LANDMARK

[50, 54, 55]: LANDMARK

81: LANDMARK

89: LANDMARK

91: LANDMARK

21) 38: 2→3

[39, 40, 46]: LANDMARK

[50, 54, 55]: LANDMARK

60: 0→1

67: 0→2

81: LANDMARK

89: LANDMARK

91: LANDMARK

22) [39, 40, 46]: LANDMARK

49: 1→2

[50, 54, 55]: LANDMARK

81: LANDMARK

89: LANDMARK

91: LANDMARK

23) 9: 1→0

33: 1→2

[39, 40, 46]: LANDMARK

53: 2→1

[50, 54, 55]: LANDMARK

75: 0→1

81: LANDMARK

89: LANDMARK

91: LANDMARK

24) [39, 40, 46]: LANDMARK

[50, 54, 55]: LANDMARK

57: 1→2

81: LANDMARK

89: LANDMARK

90: 0→1

91: LANDMARK

25) 33: 2→0

[39, 40, 46]: LANDMARK

53: 1→0

[50, 54, 55]: LANDMARK

81: LANDMARK

89: LANDMARK

91: LANDMARK

26) 38: 3→2

43: 2→0

[39, 40, 46]: LANDMARK

[50, 54, 55]: LANDMARK

64: 0→1

81: LANDMARK

87: 0→1, 2

89: LANDMARK

91: LANDMARK

27) [39, 40, 46]: LANDMARK

[50, 54, 55]: LANDMARK

59: 1, 2→3

81: LANDMARK

84: 0→1

85: 1→0

89: LANDMARK

91: LANDMARK

28) 2: 1→2

[39, 40, 46]: LANDMARK

[50, 54, 55]: LANDMARK

81: LANDMARK

89: LANDMARK

91: LANDMARK

29) 19: 0→1

[39, 40, 46]: LANDMARK

[50, 54, 55]: LANDMARK

81: LANDMARK

88: 0→1

89: LANDMARK

91: LANDMARK

30) 29: 0→1

[39, 40, 46]: LANDMARK

[50, 54, 55]: LANDMARK

65: 0→1

81: LANDMARK

89: LANDMARK

91: LANDMARK

31) 3: 0→1

4: 0→1

7: 1→4

[39, 40, 46]: LANDMARK

47: 0→1

[50, 54, 55]: LANDMARK

81: LANDMARK

89: LANDMARK

91: LANDMARK

32) [39, 40, 46]: LANDMARK

48: 0→1

[50, 54, 55]: LANDMARK

81: LANDMARK

89: LANDMARK

91: LANDMARK

33) 19: 0→1

[39, 40, 46]: LANDMARK

[50, 54, 55]: LANDMARK

81: LANDMARK

89: LANDMARK

91: LANDMARK

34) 30: 1→0

[39, 40, 46]: LANDMARK

[50, 54, 55]: LANDMARK

60: 1→2

81: LANDMARK

89: LANDMARK

91: LANDMARK

35) 3: 1→0

4: 1→0

[39, 40, 46]: LANDMARK

[50, 54, 55]: LANDMARK

81: LANDMARK

89: LANDMARK

90: 0→1

91: LANDMARK

36) 7: 4→1

8: 2→1

[39, 40, 46]: LANDMARK

[50, 54, 55]: LANDMARK

81: LANDMARK

89: LANDMARK

91: LANDMARK

37) 7: 4→2

22: 2→3

[39, 40, 46]: LANDMARK

[50, 54, 55]: LANDMARK

81: LANDMARK

89: LANDMARK

91: LANDMARK

38) 11: 1→0

[39, 40, 46]: LANDMARK

[50, 54, 55]: LANDMARK

81: LANDMARK

89: LANDMARK

91: LANDMARK

39) [39, 40, 46]: LANDMARK

48: 1→0

53: 1→0

[50, 54, 55]: LANDMARK

81: LANDMARK

89: LANDMARK

91: LANDMARK

40) 19: 1→0

[39, 40, 46]: LANDMARK

[50, 54, 55]: LANDMARK

57: 2→3

59: 2→3

63: 1→0

81: LANDMARK

89: LANDMARK

91: LANDMARK

41) [39, 40, 46]: LANDMARK

[50, 54, 55]: LANDMARK

81: LANDMARK

89: LANDMARK

91: LANDMARK

**Discrete + Continuous + GM tree (excluding landmark ‘state changes’)**

1) [39, 40, 46]: LANDMARK

[50, 54, 55]: LANDMARK

81: LANDMARK

89: LANDMARK

91: LANDMARK

2) 13: 0→1

22: 0→1

[39, 40, 46]: LANDMARK

[50, 54, 55]: LANDMARK

81: LANDMARK

89: LANDMARK

91: LANDMARK

3) 36: 0→1

[39, 40, 46]: LANDMARK

[50, 54, 55]: LANDMARK

69: 0→1

81: LANDMARK

89: LANDMARK

91: LANDMARK

4) 23: 0→1

26: 0→1

[39, 40, 46]: LANDMARK

[50, 54, 55]: LANDMARK

81: LANDMARK

89: LANDMARK

91: LANDMARK

5) 25: 0.452-0.470 → 0.409-0.446

[39, 40, 46]: LANDMARK

[50, 54, 55]: LANDMARK

81: LANDMARK

82: 0→1

89: LANDMARK

91: LANDMARK

6) 1: 0→1

11: 0.459-0.484 → 0.490

22: 1→2

25: 0.452-0.470 → 0.514

[39, 40, 46]: LANDMARK

[50, 54, 55]: LANDMARK

81: LANDMARK

83: 1→0

89: LANDMARK

91: LANDMARK

7) 8: 0.178-0.219 → 0.253-0.616

11: 0.490 → 0.686-0.800

34: 0→1

42: 1→0

43: 0.126-0.150 → 0.104-0.106

[39, 40, 46]: LANDMARK

[50, 54, 55]: LANDMARK

81: LANDMARK

89: LANDMARK

91: LANDMARK

8) [39, 40, 46]: LANDMARK

[50, 54, 55]: LANDMARK

81: LANDMARK

89: LANDMARK

91: LANDMARK

9) 9: 0→1

14: 1→2

[39, 40, 46]: LANDMARK

[50, 54, 55]: LANDMARK

80: 0→1

81: LANDMARK

89: LANDMARK

91: LANDMARK

10) 19: 0→1

22: 2→1

25: 0.514 → 0.503

[39, 40, 46]: LANDMARK

81: LANDMARK

89: LANDMARK

91: LANDMARK

92: 0→1

11) [39, 40, 46]: LANDMARK

58: 0→1

81: LANDMARK

87: 0.106-0.132 → 0.103

89: LANDMARK

91: LANDMARK

12) 42: 0→1

[39, 40, 46]: LANDMARK

51: 0→1

81: LANDMARK

89: LANDMARK

91: LANDMARK

13) 12: 0→1

17: 0→1

66: 0→1

70: 0→1

81: LANDMARK

14) 69: 2→1

81: LANDMARK

15) 5: 0→1

16: 1→0

25: 0.514 → 0.684-0.718

41: 0→1

[39, 40, 46]: LANDMARK

[50, 54, 55]: LANDMARK

81: LANDMARK

89: LANDMARK

90: 0→1

91: LANDMARK

16) [39, 40, 46]: LANDMARK

[50, 54, 55]: LANDMARK

57: 0→1

81: LANDMARK

89: LANDMARK

91: LANDMARK

17) 10: 0→1

[39, 40, 46]: LANDMARK

[50, 54, 55]: LANDMARK

79: 0→1

81: LANDMARK

87: 0.117-0.132 → 0.145

89: LANDMARK

91: LANDMARK

18) 11:0.686-0.690 → 0.729

43: 0.104-0.106 → 0.103

[39, 40, 46]: LANDMARK

[50, 54, 55]: LANDMARK

81: LANDMARK

89: LANDMARK

91: LANDMARK

19) [39, 40, 46]: LANDMARK

48: 0→1

[50, 54, 55]: LANDMARK

81: LANDMARK

89: LANDMARK

91: LANDMARK

20) 21: 0→1

29: 1→0

[39, 40, 46]: LANDMARK

[50, 54, 55]: LANDMARK

81: LANDMARK

89: LANDMARK

91: LANDMARK

21) [39, 40, 46]: LANDMARK

49: 1→2

53: 2→0

[50, 54, 55]: LANDMARK

57: 1→2

67: 0→2

81: LANDMARK

89: LANDMARK

91: LANDMARK

22) 38: 0.251 → 0.521

[39, 40, 46]: LANDMARK

[50, 54, 55]: LANDMARK

81: LANDMARK

89: LANDMARK

91: LANDMARK

23) 19: 0→1

20: 1→0

[39, 40, 46]: LANDMARK

[50, 54, 55]: LANDMARK

81: LANDMARK

89: LANDMARK

91: LANDMARK

24) 33: 0→1

38: 0.218 → 0.442-0.457

[39, 40, 46]: LANDMARK

[50, 54, 55]: LANDMARK

67: 0→2

81: LANDMARK

89: LANDMARK

90: 1→0

91: LANDMARK

25) 3: 0→1

4: 0→1

[39, 40, 46]: LANDMARK

49: 1→2

[50, 54, 55]: LANDMARK

81: LANDMARK

89: LANDMARK

91: LANDMARK

26) 43: 0.106-0.118 → 0.070

[39, 40, 46]: LANDMARK

[50, 54, 55]: LANDMARK

73: 1→2

76: 0→1

81: LANDMARK

89: LANDMARK

91: LANDMARK

27) [39, 40, 46]: LANDMARK

53: 2→1

[50, 54, 55]: LANDMARK

70: 0→1

81: LANDMARK

89: LANDMARK

91: LANDMARK

28) [39, 40,46]:

[50, 54, 55]: LANDMARK

59: 1→2

60: 0.098-0.102 → 0.125

81: LANDMARK

89: LANDMARK

91: LANDMARK

29) [39, 40, 46]: LANDMARK

[50, 54, 55]: LANDMARK

60: 0.124 → 0.236-0.267

81: LANDMARK

89: LANDMARK

91: LANDMARK

30) [39, 40, 46]: LANDMARK

48: 0→1

[50, 54, 55]: LANDMARK

81: LANDMARK

89: LANDMARK

91: LANDMARK

31) 35: 0→1

[39, 40, 46]: LANDMARK

[50, 54, 55]: LANDMARK

81: LANDMARK

89: LANDMARK

91: LANDMARK

32) 3: 1→0

4: 1→0

7: 2→1

[39, 40, 46]: LANDMARK

[50, 54, 55]: LANDMARK

75: 0→1

81: LANDMARK

89: LANDMARK

91: LANDMARK

33) 38: 0.442-0.457 → 0.077-0.319

[39, 40, 46]: LANDMARK

[50, 54, 55]: LANDMARK

81: LANDMARK

89: LANDMARK

90: 0→1

91: LANDMARK

34) 19: 1→0

[39, 40, 46]: LANDMARK

[50, 54, 55]: LANDMARK

81: LANDMARK

89: LANDMARK

91: LANDMARK

35) [39, 40, 46]: LANDMARK

48: 1→0

[50, 54, 55]: LANDMARK

81: LANDMARK

86: 0→1

89: LANDMARK

91: LANDMARK

36) 25: 0.325-0.439 → 0.113

34: 1→2

[39, 40, 46]: LANDMARK

53: 1, 2→0

[50, 54, 55]: LANDMARK

81: LANDMARK

89: LANDMARK

91: LANDMARK

37) [39, 40, 46]: LANDMARK

[50, 54, 55]: LANDMARK

81: LANDMARK

84: 0→1

87: 0.338-0.401 → 0.574-0.577

89: LANDMARK

38) 7: 1→2

[39, 40, 46]: LANDMARK

[50, 54, 55]: LANDMARK

57: 2→3

81: LANDMARK

89: LANDMARK

91: LANDMARK

39) [39, 40, 46]: LANDMARK

[50, 54, 55]: LANDMARK

57: 2→1

61: 2→1

81: LANDMARK

85: 1→0

89: LANDMARK

91: LANDMARK

40) 2: 1→2

20: 0→1

[39, 40, 46]: LANDMARK

[50, 54, 55]: LANDMARK

81: LANDMARK

89: LANDMARK

91: LANDMARK

41) 19: 0→1

[39, 40, 46]: LANDMARK

[50, 54, 55]: LANDMARK

81: LANDMARK

88: 0→1

89: LANDMARK

91: LANDMARK

**References**

**Ballew, K. L.** 1989. *A phylogenetic analysis of Phytosauria (Reptilia: Archosauria) from the Late Triassic of the western United States*. Unpublished MA thesis, University of California, Berkeley, 73 pp.

**Buffetaut, E.** 1993. Phytosaurs in time and space. *Paleontolol Lombarda Nuova serie*, **2**, 39-44.

**Butler, R. J., Rauhut, O. W., Stocker, M. R., & Bronowicz, R.** 2014. Redescription of the phytosaurs *Paleorhinus* (‘*Francosuchus*’) *angustifrons* and *Ebrachosuchus neukami* from Germany, with implications for Late Triassic biochronology. *Zoological Journal of the Linnean Society*, **170**, 155-208 DOI: 10.1111/zoj12094

**Camp, C. L.** 1930. A study of the phytosaurs with description of new material from western North America. *Memoirs of the University of California*, **10**, 1-174.

**Case, E. C.** 1920. Preliminary description of a new suborder of phytosaurian reptiles with a description of a new species of *Phytosaurus.* *The Journal of Geology*, **28**, 524-535 DOI: 10.1086/622732

**Case, E. C.** 1922. *New reptiles and stegocephalians from the Upper Triassic of western Texas* (Vol. 321). Carnegie Institution of Washington, 126 pp

**Case, E. C., & White, T. E.** 1934. Two new specimens of phytosaurs from the Upper Triassic of western Texas. *Contributions from the Museum of Paleontology, University of Michigan*, **4**, 133-142.

**Chatterjee, S.** 1974. A Rhynchosaur from the Upper Triassic Maleri Formation of India. *Philosophical Transactions of the Royal Society of London Series B*, **267**, 209-261 DOI: 10.1098/rstb.1974.0001

**Chatterjee, S.** 1978. A primitive parasuchid (phytosaur) reptile from the Upper Triassic Maleri Formation of India. *Palaeontology*, **21**, 83-127.

**Colbert, E. H.** 1947. Studies of the phytosaurs *Machaeroprosopus* and *Rutiodon*. *Bulletin of the AMNH*, **88**, 53-96, 10 pls

**Cope, E. D.** 1881. *Belodon* in New Mexico. *American Naturalist*, 15, 922-923.

**Drevermann, F.** 1918. Ein Parasuchier-Schädel aus dem schwäbischen Stubensandstein. *Bericht der Senckenbergischen Naturforschenden Gesellschaft in Frankfurt am Main*, **47**, 120-123.

**Dutuit, J. M.** 1977. *Paleorhinus magnoculus*, phytosaure du Trias supérieur de l’Atlas marocain. *Géologie Méditerranéenne*, **4**, 255-268.

**Emmons, E.** 1856. *Geological report of the midland counties of North Carolina*. Putnam. 352 pp., 14 pls DOI: 10.5962/bhl.title.34269

**Fraas, E.** 1896. *Die schwäbischen Trias-Saurier nach dem Material der Kgl.* Naturalien-Sammlung in Stuttgart zusammengestellt. Stuttgart (Schweizerbart). 18 pp.

**Gregory, J. T.** 1962. The genera of phytosaurs. *American Journal of Science*, **260**, 652-690 DOI: 10.2475/ajs.260.9.652

**Gregory, J. T.** 1972. Vertebrate faunas of the Dockum Group, Triassic, eastern New Mexico and West Texas. *New Mexico Geological Society 23rd Fall Field Conference, Guidebook*, 120-123.

**Griffin, C. T., Stefanic, C. M., Parker, W. G., Hungerbühler, A., & Stocker, M. R.** 2017. Sacral anatomy of the phytosaur *Smilosuchus adamanensis*, with implications for pelvic girdle evolution among Archosauriformes. *Journal of Anatomy*, **231**, 886-905 DOI: 10.1111/joa.12681

**Heckert, A. B., Lucas, S. G., Hunt, A. P., & Harris, J. D.** 2001. A giant phytosaur (Reptilia: Archosauria) skull from the Redonda Formation (Upper Triassic: Apachean) of east-central New Mexico. *New Mexico Geological Society Guidebook*, **52**, 171-178.

**Heckert, A. B., & Lucas, S. G.** 2003. Stratigraphy and paleontology of the lower Chinle Group (Adamanian; latest Carnian) in the vicinity of St. Johns, Arizona. *New Mexico Geological Society Guidebook*, **54**, 281-288.

**Heller, F.** 1954. Ein Parasuchier-Schädelrest aus dem Oberen Burgsandstein von Coburg. *Geologische Blätter für Nordost-Bayern und angrenzende Gebiete*, 4, 1-14.

**Huene, F. von.** 1909. Vorläufige Mitteilung über einen neuen *Phytosaurus*-Schädel aus dem schwäbischen Keuper. *Centralblatt für Mineralogie, Geologie und Paläontologie*, **1909** 583-592.

**Huene, F. von.** 1911. Beiträge zur Kenntnis und Beurteilung der Parasuchier. *Geologische und Paläontologische Abhandlungen, Neue Folge*, **10**, 67-121.

**Huene, F. von.** 1922. Neue Beiträge zur Kenntnis der Parasuchier. *Jahrbuch der Preussischen Geologischen Landesanstalt*, **42**, 146-148.

**Hungerbühler, A.** 1998. *Cranial anatomy and diversity of the Norian phytosaurs of Southwestern Germany*. Unpublished PhD thesis, University of Bristol, 464 pp.

**Hungerbuhler, A., & Hunt, A. P.** 2000. Two new phytosaur species (Archosauria, Crurotarsi) from the Upper Triassic of southwest Germany. *Neues Jahrbuch fur Geologie und Palaontologie-Monatshefte*, **8**, 467-484.

**Hungerbühler, A.** 2002. The Late Triassic phytosaur *Mystriosuchus westphali*, with a revision of the genus. *Palaeontology*, **45**, 377-418 DOI: 10.1111/1475-4983.00242

**Hungerbühler, A., Mueller, B., Chatterjee, S. & Cunningham, D. P.** 2013. Cranial anatomy of the Late Triassic phytosaur *Machaeroprosopus*, with the description of a new species from West Texas. *Earth and Environmental Science Transactions of the Royal Society of Edinburgh*, **103**, 269–312 DOI: 10.1017/S1755691013000364

**Hunt, A. P.** 1994a. The phylogeny and biochronology of the Parasuchidae (Reptilia: Archosauria). *Journal of Vertebrate Paleontology*,**14**(3), 30A DOI: 10.1080/02724634.1994.10011592

**Hunt, A P.** 1994b. *Vertebrate paleontology and biostratigraphy of the Bull Canyon Formation (Chinle Group, Upper Triassic), east-central New Mexico with revisions of the families Metoposauridae (Amphibia: Temnospondyli) and Parasuchidae (Reptilia: Archosauria).* Unpublished PhD thesis, University of New Mexico, 404 pp.

**Hunt, A. P., & Lucas, S. G.** 1989. New genotype designations for the phytosaurs *Mystriosuchus* and *Rutiodon* with a discussion of the taxonomic status of *Mystriosuchus*, *Clepsysaurus* and *Rutiodon*. Pp. 340-348 in S. G. Lucas & A. P. Hunt (eds) *Dawn of the Age of Dinosaurs in the American Southwest*. New Mexico Museum of Natural History and Science, Albuquerque.

**Hunt, A. P., & Lucas, S. G.** 1993. A new phytosaur (Reptilia: Archosauria) genus. *New Mexico Museum of Natural History & Science Bulletin*, **3**, 193-196.

**Hunt, A. P., Lucas, S. G., & Bircheff, P.** 1993. Biochronological significance of the co-occurrence of the phytosaurs (Reptilia: Archosauria) *Angistorhinus* and *Rutiodon* in the Los Esteros Member of the Santa Rosa Formation, Santa Fe County, New Mexico, USA. *New Mexico Museum of Natural History & Science Bulletin*, **3**, 203-204.

**Hunt, A. P., Lucas, S. G., & Spielmann, J. A.** 2006. Sexual dimorphism in a large brachyrostral phytosaur (Archosauria: Crurotarsi) from the Late Triassic of western North America. *New Mexico Museum of Natural History and Science Bulletin*, **37**, 563-67.

**Irmis, R. B.** 2005. The vertebrate fauna of the Upper Triassic Chinle Formation in northern Arizona. *Mesa Southwest Museum Bulletin*, **9**, 63-88.

**Kammerer, C. F., Butler, R. J., Bandyopadhyay, S., & Stocker, M. R.** 2015. Relationships of the Indian phytosaur *Parasuchus hislopi* Lydekker, 1885. *Papers in Palaeontology*, **2**, 1-23 DOI: 10.1002/spp2.1022

**Kuhn, O.** 1936. Weitere Parasuchier und Labyrinthodonten aus dem Blasensandstein des mittleren Keuper von Ebrach. *Palaeontographica Abteilung* A, **83**, 61–98.

**Langston, W.** 1949. A new species of *Paleorhinus* from the Triassic of Texas. *American Journal of Science*, **247**, 324-341 DOI: 10.2475/ajs.247.5.324

**Lees, J. H.** 1907. The skull of *Paleorhinus*: a Wyoming phytosaur. *The Journal of Geology*, **15**, 121-151 DOI: 10.1086/621382

**Li, C., Wu, X. C., Zhao, L. J., Sato, T., & Wang, L. T.** 2012. A new archosaur (Diapsida, Archosauriformes) from the marine Triassic of China. *Journal of Vertebrate Paleontology*, **32**, 1064-1081 DOI: 10.1080/02724634.2012.694383

**Long, R. A., & Murry, P. A.** 1995. *Late Triassic (Carnian and Norian) Tetrapods from the Southwestern United States: Bulletin* *4*. New Mexico Museum of Natural History and Science.

**Lucas, S. G., Heckert, A. B., Zeigler, K. E., & Hunt, A. P.** 2002. The type locality of *Belodon buceros* Cope, 1881, a phytosaur (Archosauria: Parasuchidae) from the Upper Triassic of north-central New Mexico. *New Mexico Museum of Natural History and Science Bulletin*, **21**, 189-192.

**Lydekker, R.** 1885. The Reptilia and Amphibia of the Maleri and Denwa groups. *Palaeontologia Indica, Series 1*, **1**, 1–38.

**Marsh, O. C.** 1896. A new belodont reptile (*Stegomus*) from the Connecticut River sandstone. *American Journal of Science*, **7**, 59-62.

**Mateus, O., Butler, R. J., Brusatte, S. L., Whiteside, J. H., & Steyer, J. S.** 2014. The first phytosaur (Diapsida, Archosauriformes) from the Late Triassic of the Iberian Peninsula. *Journal of Vertebrate Paleontology*, **34**, 970-975 DOI: 10.1080/02724634.2014.840310

**McCormack L, Parker WG.** 2017. A new occurrence of the phytosaur (Archosauriformes, Phytosauria) *Pravusuchus hortus* from the Monitor Butte Memeber (Upper Triassic; Chinle Formation) of Utah. *Journal of Vertebrate Paleontology Program and Abstracts, 2017*, 161 pp

**McGregor, J. H.** 1906. The Phytosauria, with especial reference to *Mystriosuchus* and *Rhytidodon*. *Memoirs of the American Museum of Natural History*, **9**, 27-101, 5 pls

**Mehl, M. G.** 1913. *Angistorhinus*, a new genus of Phytosauria from the Trias of Wyoming. *The Journal of Geology*, **21**, 186-191 DOI: 10.1086/622049

**Mehl, M. G.** 1922. A new phytosaur from the Trias of Arizona. *The Journal of Geology*, **30**, 144-157 DOI: 10.1086/622860

**Mehl, M. G.** 1928. The Phytosauria of the Wyoming Triassic. *Journal of the Denison University Laboratories, Denison University*, **23**, 141–172.

**Meyer, H. von.** 1860. Briefliche Mittheilung an Prof. Bronn. *Neues Jahrbuch fur Mineralogie, Geognosie, Geologie und Petrefakten-Kunde*, 556-560.

**Meyer, H. von.** 1861. Reptilien aus dem Stubensandstein des oberen Keupers. *Palaeontographica*, **7**, 253-351, pls 28-47.

**Meyer, H. von.** 1863. Der Schädel des *Belodon* aus dem Stubensandstein des oberen Keupers. *Palaeontographica*, **10**, 227-246, pls 38-42.

**Meyer, H. von.** 1865. Reptilien aus dem Stubensandstein des oberen Keupers. *Palaeontographica*, **14**, 99-124, pls 23-29.

**Parker, W. G.** 2002. Correlation of locality numbers for vertebrate fossil sites in Petrified Forest National Park, Arizona. *New Mexico Museum of Natural History and Science Bulletin*, **21**, 37-42.

**Parker, W. G., & Irmis, R. B.** 2006. A new species of the Late Triassic phytosaur *Pseudopalatus* (Archosauria: Pseudosuchia) from Petrified Forest National Park, Arizona. *Museum of Northern Arizona Bulletin*, **62**, 126-143.

**Parker, W. G., & Martz, J. W.** 2011. The Late Triassic (Norian) Adamanian–Revueltian tetrapod faunal transition in the Chinle Formation of Petrified Forest National Park, Arizona. *Earth and Environmental Science Transactions of the Royal Society of Edinburgh*, **101**, 231-260.

**Pinna, G.** 1987. I nuovi Lagerstaetten fossili del Triassico Italiano. *Le Scienze*, **224**, 62-70.

**Sereno, P. C.** 1991. Basal archosaurs: phylogenetic relationships and functional implications. *Journal of Vertebrate Paleontology*, **11**(S4), 1-53 DOI: 10.1080/02724634.1991.10011426

**Spielmann, J. A., & Lucas, S. G.** 2012. Tetrapod Fauna of the Upper Triassic Redona Formation East-central New Mexico: The Characteristic Assemblage of the Apachean Land-vertebrate Faunachron*. New Mexico Museum of Natural History and Science Bulletin*, **55**, 1-119.

**Stocker, M. R.** 2010. A new taxon of phytosaur (Archosauria: Pseudosuchia) from the Late Triassic (Norian) Sonsela Member (Chinle Formation) in Arizona, and a critical reevaluation of *Leptosuchus* Case, 1922. *Palaeontology*, **53**, 997-1022 DOI: 10.1111/j.1475-4983.2010.00983.x

**Stocker, M. R.** 2012. A new phytosaur (Archosauriformes, Phytosauria) from the Lot's Wife beds (Sonsela Member) within the Chinle Formation (Upper Triassic) of Petrified Forest National Park, Arizona. *Journal of Vertebrate Paleontology*, **32**, 573-586 DOI: 10.1080/02724634.2012.649815

**Stocker, M. R.** 2013. A new taxonomic arrangement for *Paleorhinus scurriensis*. *Earth and Environmental Science Transactions of the Royal Society of Edinburgh*, **103**, 1-13 DOI: 10.1017/S1755691013000340

**Stocker, M. R., & Butler, R. J.** 2013. Phytosauria. *Geological Society, London, Special Publications*, **379**, 91-117 DOI: 10.1144/SP379.5

**Stocker, M. R., Zhao, L. J., Nesbitt, S. J., Wu, X. C., & Li, C.** 2017. A Short-Snouted, Middle Triassic Phytosaur and its Implications for the Morphological Evolution and Biogeography of Phytosauria. *Scientific reports*, **7**, 46028 DOI: 10.1038/srep46028

**Williston, S. W.** 1904. Notice of some new reptiles from the Upper Trias of Wyoming. *The Journal of Geology*, **12**, 688-697 DOI: 10.1086/621190

**Witmer, L. M.** 1997. The evolution of the antorbital cavity of archosaurs: a study in soft-tissue reconstruction in the fossil record with an analysis of the function of pneumaticity. *Journal of Vertebrate Paleontology*, **17**(S1), 1-76 DOI: 10.1080/02724634.1997.10011027

**Zeigler, K. E., Lucas, S. G., & Heckert, A. B.** 2002. A phytosaur skull from the upper Triassic Snyder Quarry (Petrified Forest Formation, Chinle Group). *Upper Triassic Stratigraphy and Paleontology: Bulletin*, **21**, 171-178.

**Zeigler, K. E., Lucas, S. G., & Heckert, A. B.** 2003a. Variation in the Late Triassic Canjilon quarry (Upper Chinle Group, New Mexico) phytosaur skulls: a case for sexual dimorphism. *Paläontologische Zeitschrift*, **77**, 341 DOI: 10.1007/BF03006946

**Zeigler, K. E., Heckert, A. B., & Lucas, S. G.** 2003b. Phytosaur (Archosauria: Parasuchidae) cranial and mandibular material from the Upper Triassic Snyder quarry (Petrified Forest Formation, Chinle Group). *New Mexico Museum of Natural History and Science* *Bulletin*, **24**, 81-88.
