# Supplementary figures and images for: A new phylogenetic analysis of Phytosauria (Archosauria: Pseudosuchia) with the application of continuous and geometric morphometric character coding
Source: PeerJ. 2018 Dec 10;6:e5901. doi: 10.7717/peerj.5901 (PMC6292387; doi:10.7717/peerj.5901)

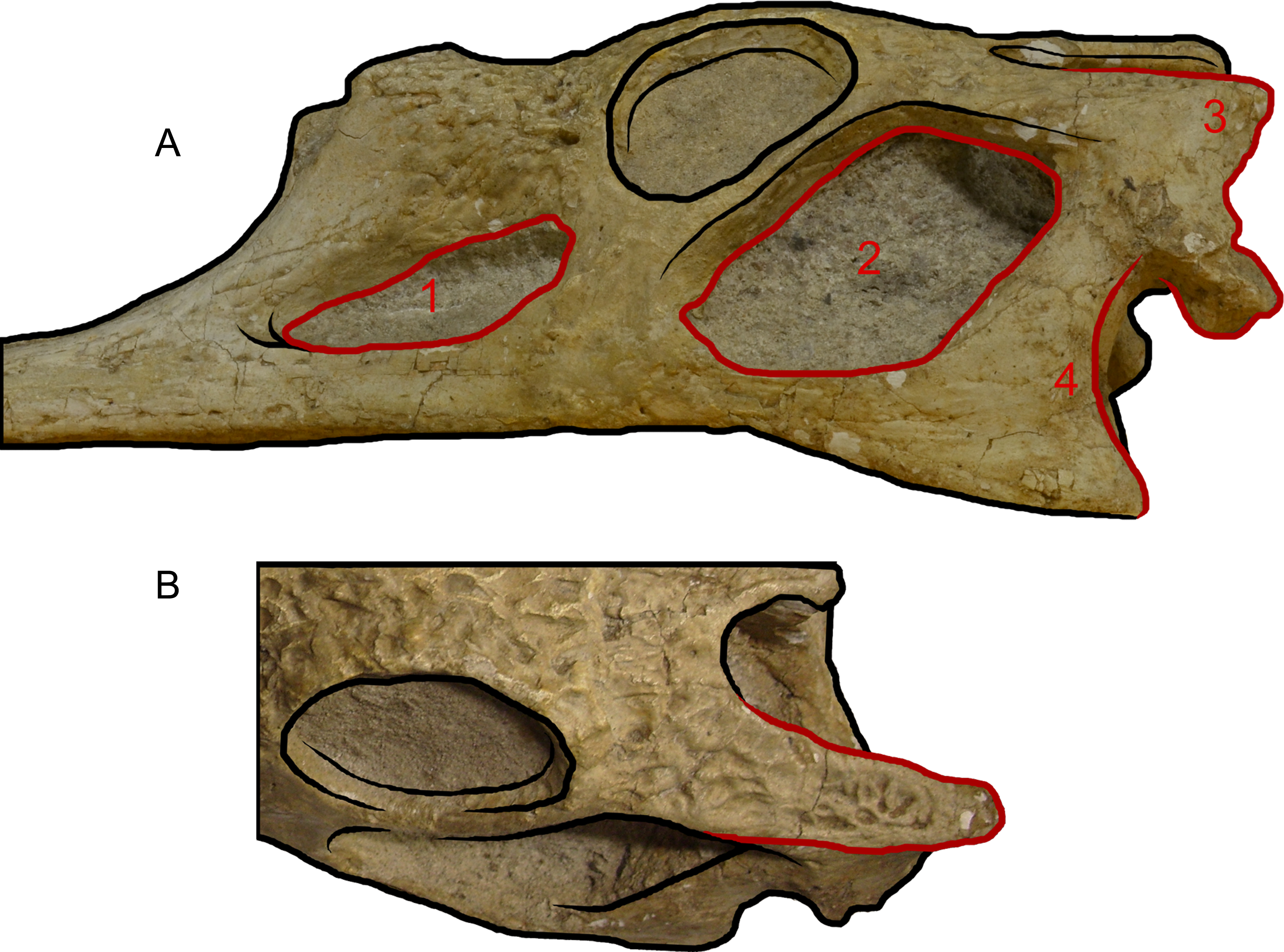

Supplement: Supplemental Information 2 — Pathways along which semilandmarks were plotted are drawn in red; these correspond to (A) 1 shape of the antorbital fenestra, 2 shape of the lateral temporal fenestra, 3 shape of the posterior process of the squamosal in lateral view, 4 posterior curvature of the quadrate and (B) shape of the postorbital/squamosal bar and the posterior process of the squamosal in dorsal view. All photographs were taken by Andrew Jones. [file peerj-06-5901-s002.png]

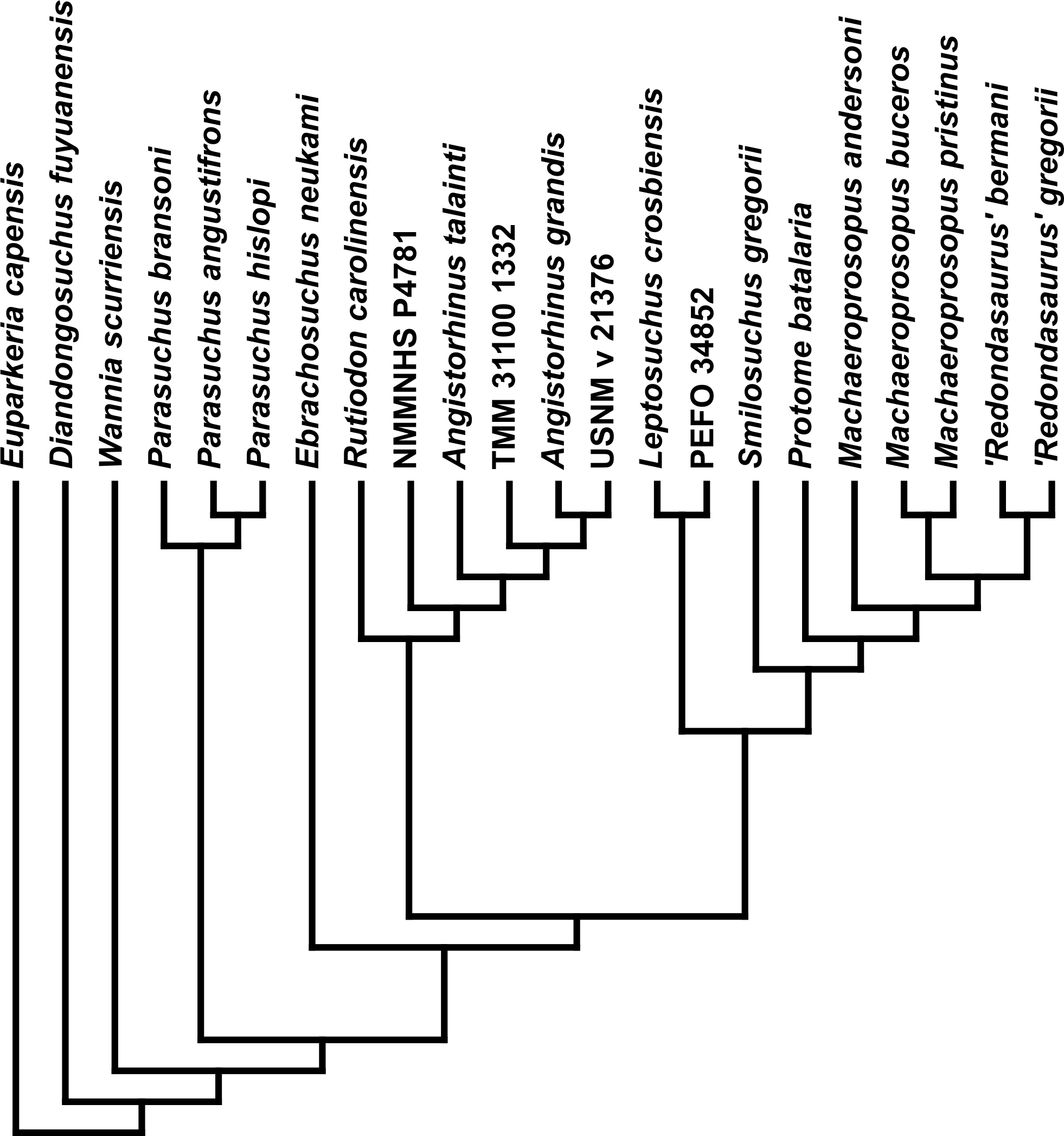

Supplement: Supplemental Information 3 — Taxa expressing conflicting relationships have been pruned. [file peerj-06-5901-s003.png]
