# Supplementary material for: A new phylogenetic analysis of Phytosauria (Archosauria: Pseudosuchia) with the application of continuous and geometric morphometric character coding
Source: PeerJ. 2018 Dec 10;6:e5901. doi: 10.7717/peerj.5901 (PMC6292387; doi:10.7717/peerj.5901)
Supplement: Supplemental Information 4 — Also included for continuous and GM data are the quantities of discrete characters that correspond to each data type, and the proportion of missing data in those discrete versions of continuous/GM characters. [file peerj-06-5901-s004.docx]

| Character type | Number of characters  *[Number of discretely scored counterparts]* | Proportion of missing data  *[Proportion missing in discretely scored counterparts]* |
| --- | --- | --- |
| Discrete | 94 *[N/A]* | 32.5% *[N/A]* |
| Continuous | 10 *[10]* | 34.3% *[31.7%]* |
| GM (Landmark) | 5 *[9]* | 41.4% *[38.4%]* |
