# Supplementary material for: A new phylogenetic analysis of Phytosauria (Archosauria: Pseudosuchia) with the application of continuous and geometric morphometric character coding
Source: PeerJ. 2018 Dec 10;6:e5901. doi: 10.7717/peerj.5901 (PMC6292387; doi:10.7717/peerj.5901)
Supplement: Supplemental Information 5 [file peerj-06-5901-s005.docx]

| OTUs | Missing discrete character scores | Percentage missing data |
| --- | --- | --- |
| *Machaeroprosopus_lottorum* | 5 | 5.3 |
| *Machaeroprosopus_pristinus* | 5 | 5.3 |
| *Mystriosuchus_planirostris* | 7 | 7.4 |
| *Nicrosaurus_kapffi* | 7 | 7.4 |
| *Machaeroprosopus_mccauleyi* | 8 | 8.5 |
| *Machaeroprosopus_buceros* | 9 | 9.6 |
| *Nicrosaurus_meyeri* | 10 | 10.6 |
| *Angistorhinus_talainti* | 13 | 13.8 |
| *Smilosuchus_gregorii* | 13 | 13.8 |
| *Mystriosuchus_westphali* | 14 | 14.9 |
| *Leptosuchus_crosbiensis* | 17 | 18.1 |
| *Parasuchus_angustifrons* | 17 | 18.1 |
| *Parasuchus_bransoni* | 17 | 18.1 |
| *Redondasaurus_gregorii* | 19 | 20.2 |
| *Smilosuchus_adamanensis* | 19 | 20.2 |
| *Ebrachosuchus_neukami* | 21 | 22.3 |
| *Rutiodon_carolinensis* | 22 | 23.4 |
| *Pravusuchus_hortus* | 23 | 24.5 |
| *Parasuchus_hislopi* | 24 | 25.5 |
| *Leptosuchus_studeri* | 25 | 26.6 |
| *Redondasaurus_bermani* | 27 | 28.7 |
| NMMNHS_P4256 | 28 | 29.8 |
| *Euparkeria_capensis* | 28 | 29.8 |
| *Smilosuchus_lithodendrorum* | 29 | 30.9 |
| TMM_31100_1332 | 29 | 30.9 |
| *Paleorhinus_sawini* | 31 | 33.0 |
| PEFO_34852 | 31 | 33.0 |
| NMMNHS_P31094 | 32 | 34.0 |
| USNM_v_17098 | 38 | 40.4 |
| *Diandongosuchus_fuyuanensis* | 39 | 41.5 |
| *Machaeroprosopus_andersoni* | 44 | 46.8 |
| *Angistorhinus_grandis* | 46 | 48.9 |
| *Protome_batalaria* | 48 | 51.1 |
| NHMW_1986_0024_0001 | 48 | 51.1 |
| *Phytosaurus_doughtyi* | 49 | 52.1 |
| *Coburgosuchus_goeckeli* | 50 | 53.2 |
| *Wannia_scurriensis* | 50 | 53.2 |
| USNM_v_21376 | 57 | 60.6 |
| MB.R._2747 | 59 | 62.8 |
| *Machaeroprosopus_zunii* | 65 | 69.1 |
| *Machaeroprosopus_jablonskiae* | 66 | 70.2 |
| NMMNHS_P4781 | 74 | 78.7 |
| *Paleorhinus_parvus* | 79 | 84.0 |
